# MIF-Dependent Control of Tumor Immunity

**DOI:** 10.3389/fimmu.2020.609948

**Published:** 2020-11-25

**Authors:** Jordan T. Noe, Robert A. Mitchell

**Affiliations:** ^1^ Department of Biochemistry and Molecular Genetics, University of Louisville, Louisville, KY, United States; ^2^ J.G. Brown Cancer Center, University of Louisville, Louisville, KY, United States; ^3^ Department of Surgery, Division of Immunotherapy, University of Louisville, Louisville, KY, United States; ^4^ Department of Microbiology and Immunology, University of Louisville, Louisville, KY, United States

**Keywords:** migration inhibitory factor, cytokines, tumor immunity, immunotherapy, macrophages, lymphocytes, dendritic cells, immune evasion

## Abstract

Initially identified as a T lymphocyte-elicited inhibitor of macrophage motility, macrophage migration inhibitory factor (MIF) has since been found to be expressed by nearly every immune cell type examined and overexpressed in most solid and hematogenous malignant cancers. It is localized to both extracellular and intracellular compartments and physically interacts with more than a dozen different cell surface and intracellular proteins. Although classically associated with and characterized as a mediator of pro-inflammatory innate immune responses, more recent studies demonstrate that, in malignant disease settings, MIF contributes to anti-inflammatory, immune evasive, and immune tolerant phenotypes in both innate and adaptive immune cell types. This review will summarize the studies describing MIF in tumor-specific innate and adaptive immune responses and attempt to reconcile these various pleiotropic functions in normal physiology.

## Introduction

Tumor immune responses are shaped and characterized by a number of factors (1) including mutational burden, inflammatory response, differential expression of cytokines and chemokines, and the presence and/or activation of both tumoral and stromal immune suppressive checkpoint ligands and receptors.

Originally identified over 50 years ago as a secreted lymphocyte product associated with macrophage-dependent delayed-type hypersensitivity (2, 3), MIF has become one of the most enigmatic regulators of innate and adaptive immune responses. Following its initial cDNA cloning in the late 1980s (4), further characterization studies identified MIF’s expression in myeloid lineage cells (5) and discovered its functional importance in driving innate immune responses [reviewed in (6)]. Although MIF was first discovered in the early 1960s, the first description of a functional role for MIF in facilitating T cell responses came more than 30 years later (7).

Since these early findings, MIF has been shown to be involved—either as an autocrine or paracrine-acting cytokine—in functional phenotypes associated with innate myeloid, neutrophil, gamma delta (*γ*δ) T cell and adaptive Th1, Th2, Th17, NKT, and B cells. Because each of these cell types influences the initiation, growth, progression, and/or metastatic dissemination of tumors, we will attempt to summarize how MIF shapes tumor-associated immune responses focusing on individual cell effectors and their relative contributions to pro/anti-tumor immunity.

## MIF’s Mechanisms of Action

**Figure 1:**
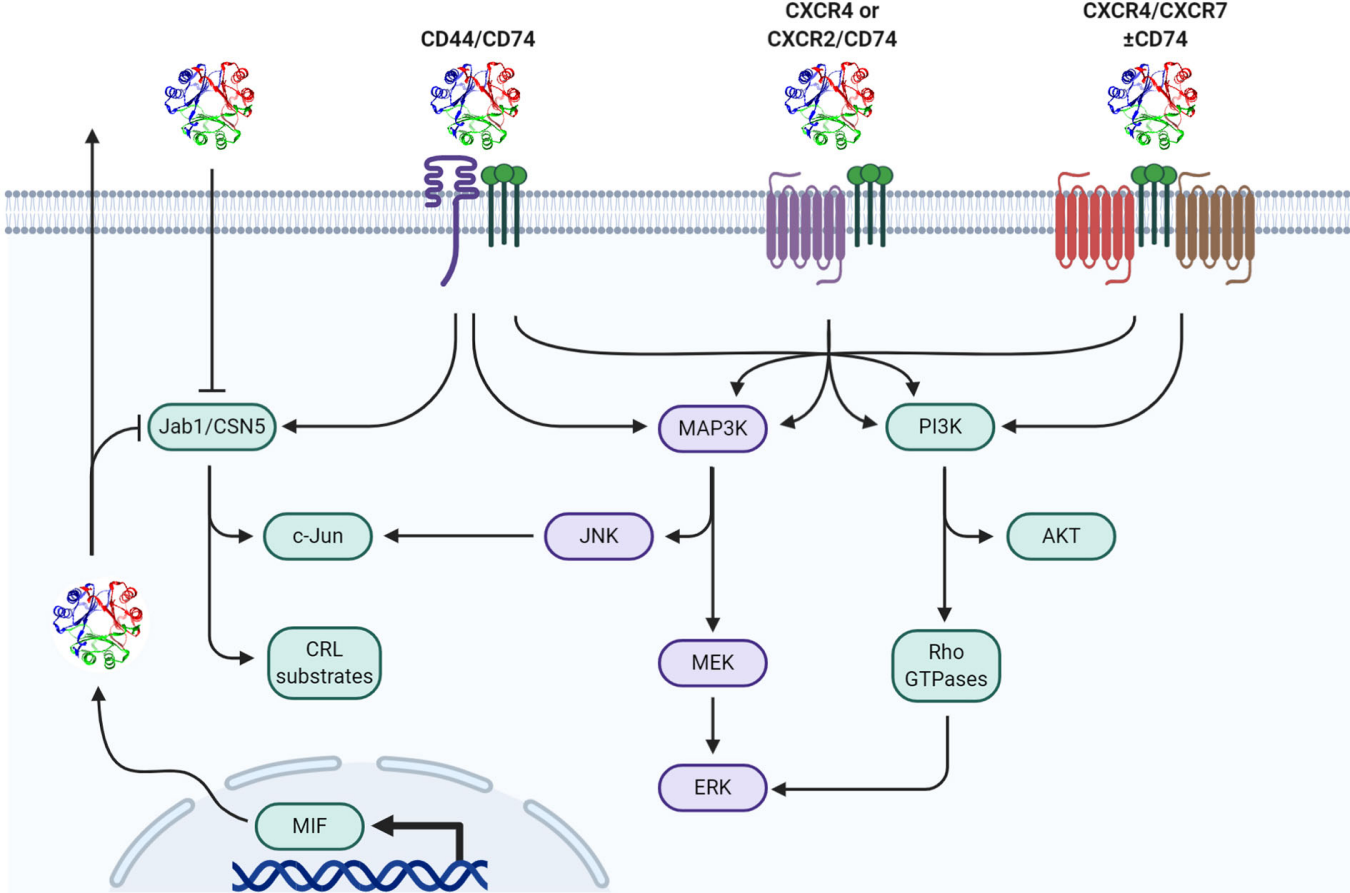
Mechanisms of MIF signal transduction. Paracrine/autocrine extracellular MIF or endogenously produced cytosolic MIF functionally interacts with cytosolic Jab1/CSN5 resulting in differential cullin-ring ligase (CRL) substrate proteasomal degradation and/or c-Jun phosphorylation/AP-1 activation. Extracellular MIF independently interacts with CD74 in hetero-complex with CD44, CXCR2, CXCR4, and/or CXCR7 to initiate downstream MAPK and/or PI3K pathway effectors.

MIF elicits bio-actions *via* both extracellular and intracellular mechanisms (**Figure 1**). Prototypical outside-in signaling occurs by extracellular MIF binding to receptor/co-receptor complexes on the cell surface while intracellular MIF can act in a receptor-independent manner by physically interacting with various intracellular proteins and enzymes thereby modifying their specific effector functions [reviewed in (8)]. It is likely that MIF’s site of action—either extracellular or intracellular—as well as which receptor/co-receptor or intracellular protein/enzyme MIF interacts with, is ultimately responsible for specific phenotypes elicited by MIF.

MIF expression and secretion is elevated in most solid and hematogenous cancers and high MIF expression is a negative prognostic indicator in several cancer types (9). The extent of MIF expression is dependent on the tumor tissue type, stage, and grade among other factors (10). For example, intratumoral MIF is increased—versus normal tissue—three- and five-fold in endometrial carcinoma and non-small cell lung carcinoma (11, 12), respectively, ten times in hepatocellular carcinoma (13) and sixty times in colorectal cancer (14). In hepatocellular carcinoma, a positive correlation was also identified between intratumoral MIF and plasma MIF, suggesting that high tumor-associated MIF expression may drive higher circulating levels of soluble MIF (13).

The current understanding by which MIF gets into or out of a cell is rather limited and the findings so far suggest atypical features. Intracellular MIF can be stored in the cytosol or secreted into the extracellular space. As MIF does not have an N-terminal signal peptide required for the classical ER/Golgi-dependent secretory pathway, MIF is instead secreted in a non-classical protein export route through ATP binding cassette transporter subfamily 1 (ABCA1) (15). Additionally, intracellular pools of MIF have also been suggested to be generated, at least in part, through cellular uptake *via* clathrin-mediated endocytosis with subsequent localization in lysosomal and cytosolic compartments (16, 17). While the exact pathways involved in how endocytosed MIF crosses endosomal or other vesicular membranes remains enigmatic (18), cellular uptake studies indicate that exogenous MIF is taken up by both immune and non-immune cells with subsequent interactions with cytosolic binding partners (16).

In addition to cancer cells, MIF is upregulated in myeloid and lymphocyte lineage cell types in response to various activating ligands as well as DAMPs (19), PAMPs (20, 21), and environmental metabolic changes (8, 22). Once secreted, MIF can signal in either a paracrine or autocrine fashion by binding to transmembrane receptors leading to intracellular transduction cascades (22, 23). The Bucala group originally identified CD74, the invariant chain of the major histocompatibility complex II (MHCII), to be a primary cognate receptor for MIF (24). Extracellular binding of MIF to cell surface CD74 initiates signal transduction through the ERK MAP kinase cascade resulting in cellular proliferation and prostaglandin E2 (PGE_2_) production (24). Additional studies suggest that following CD74 receptor binding, MIF undergoes endocytosis to sustain this signal transduction cascade—while still in the endosome—through CD74-dependent recruitment of β-arrestin1 and subsequent ERK activation (17). Because CD74 does not possess a cytoplasmic tail capable of instigating downstream signaling, MIF-bound CD74 forms a hetero-complex with CD44 which then allows for canonical ERK Map Kinase pathway activation (25).

In addition to signaling through CD74/CD44 complexes, MIF has also been shown to be a non-cognate ligand for the chemokine receptors CXCR2, CXCR4, and CXCR7 and acts as a chemokine-like molecule resulting in monocyte activation of Gαi- and integrin-dependent adhesion and recruitment (26–28). Given that these receptors are variably expressed on numerous immune cell types implicated in different aspects of tumor immune responses, the effector function(s) and biological activities elicited by extracellular MIF are likely highly dependent on signals stemming from the microenvironment and immune landscape within the tumor stroma that control relative expression levels of each.

In addition to its extracellular receptor-dependent functions, cytosolic MIF binds to several different intracellular proteins to modulate their biological activities. The best characterized of these intracellular effectors is the COP9-signalosome subunit 5 (CSN5), which is an important determinant of cullin-dependent protein turnover (29, 30). CSN5 has also been shown to dissociate from the CSN complex where it can facilitate transactivation of c-Jun transcription and, in this context, is also referred to as Jun-activation domain-binding protein (Jab1) (31). Bernhagen’s group first identified that MIF negatively regulates the activity of cytosolic Jab1 on both steady-state and stimulus-induced AP-1-dependent transcription (18, 32). Given that AP-1 is associated with activation of pro-inflammatory responses in numerous immune cell types (33, 34), an anti-inflammatory/immunosuppressive role for MIF might be expected when intracellular MIF concentrations are at sufficient levels to functionally inhibit Jab1/CSN5. In contrast to intracellular MIF, extracellular MIF/CD74 interaction is shown to functionally *activate* c-Jun phosphorylation and increase AP-1-dependent transcription (35–37) so it is conceivable, if not likely, that the relative balance between extracellular and intracellular MIF levels in a tumor microenvironment or the circulation at any given time dictates the ensuing MIF-associated phenotype.

In addition to its AP-1 regulatory activities through Jab1 binding, MIF also modulates Jab1/CSN5-dependent ubiquitylation/proteasomal degradation of a variety of proteins. These include p27^Kip1^ (18), Cdc25A (38), E2F family members (38), and, more recently, HIF-1α (39, 40). In hypoxic tissues, such as the tumor microenvironment, there is a reciprocally synergistic relationship between MIF and HIF-1α; hypoxia-driven HIF-1α stabilization and subsequent transcriptional regulation promotes an enhanced expression of MIF (41). This ensuing increase in MIF promotes interaction with CSN5 to prevent the degradation of HIF-1α—either by sterically preventing HIF1α prolyl-564 hydroxylation by direct binding or by binding to VHL and preventing the recognition/ubiquitination of hydroxylated HIF1α that is targeted for proteasomal degradation (42)—which in turn amplifies the transcriptional response of HIF-1α (39). Given the important role of HIF-1α in regulating the phenotypes and relative differentiation/maturation of multiple different immune cell types (43), intracellular MIF-mediated HIF-1α stability may be a centrally important mechanism of action responsible for several of the MIF-associated pro/anti-tumor immune phenotypes described below.

## Tumor-Associated Macrophages

Tumor-associated macrophages (TAMs) are innate immune cells of the myeloid lineage that reside within the tumor following infiltration as either immature monocyte/macrophages (44, 45) or monocytic myeloid-derived suppressor cells (MDSCs) (46). TAMs are increasingly being recognized as central determinants in the shaping of the angiogenic, matricellular, and immune composition of tumor microenvironments [reviewed in (35–37)]—so much so that there are now numerous active clinical trials seeking to modify or disrupt TAM-dependent pro-tumorigenic phenotypes (47).

TAMs have a “yin-yang” relationship in tumor initiation and progression depending on both the tumor stage and the phenotype of the TAM. Intratumoral TAM polarization is dictated by the composition of tumor-derived *vs.* immune cell-derived cytokines (48, 49), growth factors (50, 51), oxygenation (52, 53), and metabolic substrate composition (54, 55) among many other factors. Combined, these factors govern whether TAMs are differentially polarized towards a more pro-tumor phenotype or anti-tumor phenotype although, in reality, there is a broad spectrum of TAM activation/polarization profiles across different tumors and even within the same lesions (56). Despite this phenotypic heterogeneity, the classical M2 and M1 TAM phenotype nomenclature can be useful in denoting anti-inflammatory, pro-tumor *vs.* inflammatory, anti-tumor behaviors, respectively (57).

“M1”-TAMs have an inflammatory/immunostimulatory phenotype as they can activate adaptive immune responses and produce inflammatory biomolecules (e.g. reactive oxygen and nitrogen species—ROS and NOS), lipids (e.g. prostaglandins, leukotrienes), and cytokines (e.g. IL-1β, TNF-α, IL-6). During tumor initiation, early-stage metaplasia, and ensuing cancer-related inflammation, M1-TAMs accumulate and promote the elicitation of an inflammatory, anti-tumor response *via* phagocytosis of tumor cells (58–60), tumor-antigen presentation (61, 62), and production of inflammatory biomolecules and cytokines (63, 64). Conversely, anti-inflammatory/immunosuppressive “M2” TAMs promote wound healing and resolution of chronic inflammatory responses that would otherwise drive tumor initiation (65), but in the context of established tumors, M2-TAMs promote evasion of anti-tumor immunity (66–72), *de novo* neoangiogenesis (73, 74), and extracellular matrix remodeling (75).

Classically, MIF is best known as a centrally important driver of local and systemic inflammation. Although MIF was initially identified as a product of activated T cells that acts in a paracrine manner to modulate the relative motility of myeloid lineage cells (3), subsequent studies found that macrophages are also an abundant and important source of MIF (5). MIF expression/activity in macrophages has been implicated in the pathogenesis of numerous inflammatory conditions including bacterial sepsis (20, 76–78), rheumatoid arthritis (79, 80), acute respiratory distress syndrome (ARDs) (81, 82), and atherosclerosis (79, 83). Consistent with these findings, MIF is instrumental in driving maximal inflammation associated with carcinogenesis and early-stage hyperplasia/carcinoma (84). This is especially true for inflammatory colitis, which is an exceptionally common pre-condition/pre-requisite for colorectal adenoma and adenocarcinoma progression (85). Macrophage MIF is transcriptionally induced and secreted by pro-inflammatory cytokines, TNF-α, and IFN-γ (5), and loss or inhibition of MIF in differentiated macrophages exposed to *E. coli* lipopolysaccharide (LPS) or *S. aureus* enterotoxin results in substantially reduced levels of TNF-α, IL-6, iNOS, and Cox-2 (86, 87).

The first studies to suggest that MIF may also be involved in governing the activation states of more “M2”-like macrophages came from studies that identified non-small cell lung carcinoma (NSCLC) cell-derived MIF is responsible for increased angiogenic activity of co-cultured human monocytes (88). These studies concluded that paracrine acting, tumor-derived MIF is responsible for initiating monocyte/macrophage-dependent angiogenesis and, likely, subsequent tumor progression. Macrophage-derived MIF was later reported to both sufficient and necessary for driving the angiogenic contribution of bone-marrow-derived macrophages to teratoma formation in mice suggesting a dominant functional role for monocyte/macrophage-derived MIF in M2 macrophage functional polarization (89). These findings were later confirmed using mouse models of both primary and metastatic melanoma in MIF^+/+^ and MIF^−/−^ mice (90). In these studies, macrophage-derived MIF was necessary for maximal angiogenic growth factor expression in M2 alternatively activated macrophages and required for the T cell immunosuppressive capacity of melanoma-polarized TAMs. Importantly, both MIF-deficient and MIF small molecule inhibitor 4-IPP-treated TAMs were found to spontaneously revert to an M1-like polarization profile (90, 91). Combined, these findings indicate that loss or inhibition of MIF in solid tumor settings very efficiently re-polarizes intratumoral TAMs from an immunosuppressive/angiogenic pro-tumor phenotype to an immunostimulatory/non-angiogenic anti-tumor phenotype (90).

Although a unifying mechanism that explains how MIF contributes to these seemingly divergent M1 (inflammatory/immune-stimulatory) and M2 (anti-inflammatory/immune-suppressive) macrophage phenotypes is still lacking but one potential explanation could be that MIF provides an amplification or general activation phenotype in macrophages that acts to simply support generalized M1 or M2 gene expression patterns. This could be through promoting metabolic, transcriptional, and/or epigenetic pathways that broadly contribute to the general activation properties of infiltrating macrophages. It is also possible that differential expression of MIF receptors governed by microenvironmental polarization cues could explain the M1 *vs.* M2 effects of MIF. While further investigations will help to elucidate these mechanistic details, it is becoming increasingly evident that MIF plays an important regulatory role in governing TAM-dependent tumor initiation, progression, and metastatic disease phenotypes.

## Myeloid-Derived Suppressor Cells

Myeloid-derived suppressor cells (MDSCs) are a heterogeneous population of highly suppressive immature myeloid cells found in both the circulation and intratumoral space. Despite the name, there are at least two different sources of MDSCs in human cancer patients—the first arises from polymorphonuclear (PMN) bone marrow precursors (PMN-MDSCs) and the second from monocytic bone marrow precursors (M-MDSCs) (please see comprehensive MDSC reviews (92–94)).

MIF was first reported to promote MDSC functional accumulation as a consequence of breast cancer cell secretion and paracrine activity towards M-MDSC differentiation in mouse models of breast cancer (95). shRNA knockdown of MIF in murine breast cancer cell lines diminished primary tumor outgrowth and dramatically reduced numbers of lung micro-metastases in immunocompetent Balb/c mice but not in immune-deficient SCID mice. This anti-tumor phenotype associated with MIF-deficient 4T1 breast cancer tumors was attributed to the elicitation of significantly fewer intratumoral M-MDSCs in both the *in vivo* breast cancer models and in an *in vitro* model of M-MDSC differentiation. Interestingly, reconstitution of shRNA-resistant wildtype MIF largely reverts the slow growth and metastases-resistant phenotype of 4T1 MIF shRNA knockdown cells and restores M-MDSC numbers while an enzymatically inactive MIF mutant construct (MIF-P2G) does not (95).

An additional example of paracrine-acting MIF driving MDSC phenotypes came from the Lathia group which identified that glioblastoma (GBM) cancer stem cell (CSC)-secreted MIF increases MDSC immune suppressive activity in an arginase 1-dependent manner (96). Interestingly, only GBM CSCs—but not non-stem GBM cells—were responsible for this MIF-dependent MDSC phenotype even though MIF was expressed (albeit variably and higher in CSCs) in both cell types. In a follow-up study, infiltrating M-MDSCs were shown to highly express the cognate MIF receptor, CD74, while G-MDSCs primarily express the non-cognate CXCR2 receptor and exhibit only minimal tumor infiltration (97). Importantly, Ibudilast—which inhibits MIF enzymatic activity and MIF:CD74 interactions (98)—reduces MDSC function and increases CD8^+^ T cell infiltration in mouse models of GBM (97).

These data are consistent with our lab’s finding that MIF-stromal deficiency and the small molecule MIF enzymatic inhibitor, 4-IPP (99), reduce melanoma primary and metastatic tumor growth in a manner that coincides with reduced M-MDSC and PMN-MDSC (aka, G-MDSCs)-dependent immune suppression (90). An important difference, however, is that *stromal* MIF—as opposed to tumor cell or CSC-derived MIF—is responsible for maximally driving M-MDSC and G-MDSC immune-suppressive activities despite the very high MIF expression/secretion still present in the melanoma cell lines used for the tumor implantation studies (90). This requirement for stromal MIF in promoting tumor burden phenotypes is consistent with other reports showing that loss of stromal MIF reduces MDSC accumulation and/or tumor growth in *de novo* oral carcinogenesis (100) and human melanoma (101). It is presently unclear why different syngeneic tumor cell lines have differential tumor cell MIF requirements but it seems plausible that endogenous, autocrine acting myeloid cell MIF and paracrine acting tumor cell MIF are both capable of promoting MDSC differentiation and immune suppression (101). However, the fact that MIF P2G mutants and 4-IPP-mediated MIF inhibition (4-IPP specifically interacts with and disrupts the Pro-2 in MIF (99)) abolishes MIF-dependent effects on MDSCs (90, 95) suggests that the cognate MIF receptor CD74—which can still interact with and signal by MIF-P2G mutants (102) and 4-IPP-inhibited MIF (103)—is not centrally involved.

More recent studies revealed that MDSC-derived autocrine MIF is necessary for maximal immunosuppression of primary M-MDSCs isolated from the peripheral blood of stage III-IV metastatic melanoma patients (101). 4-IPP phenocopies its mouse MDSC inhibitory effects in human CD14^+^/HLA-DR^low^ monocytic MDSCs and suppresses antigen-independent proliferation of autologous T cells and IFN-γ production. Perhaps more importantly, exposure of patient MDSCs to 4-IPP for longer periods of time results in reductions in the expression of MDSC markers CD14 and PD-L1 and a simultaneous increase in the expression of dendritic cell (DC) markers CD80, CD83, CD86, and CD40 (101). Notably, this apparent induction of MDSC→DC phenotypic differentiation by 4-IPP results in functional DC maturation/activation and subsequent antigen-dependent T cell activation—an effect that is phenocopied by MIF^−/−^ MDSCs (101).

Other studies identifying a contributory role for MIF in MDSC-dependent tumor progression include a finding that an IG-CDR-based peptide that disrupts MIF-CD74 signaling is sufficient to reduce M-MDSC accumulation in metastatic melanoma lesions (104). This supports the GBM study suggesting a MIF-CD74 specific function in driving M-MDSC intratumoral accumulation and, likely, immune suppression (97). Also consistent with a paracrine function for tumor-derived MIF, M-MDSC differentiation is induced by human pediatric rhabdomyosarcoma and multiple myeloma cell lines in a MIF-dependent manner (105, 106) and that MIF induces the recruitment and accumulation of M-MDSCs into bladder cancer lesions (107). Interestingly, this study—in contrast to GBM and metastatic melanoma studies—found that the non-cognate MIF receptor CXCR2 was responsible for this activity.

Collectively, these studies clearly point to a centrally important role for MIF in driving tumor-associated MDSC phenotypes. What is far less clear is the mechanism and respective sources of MIF that are used to maintain these MDSC activities which range from homing, motility, expansion, differentiation, and immune-suppressive function. More studies are needed to delineate the respective mechanism(s) for MIF in driving these MDSC phenotypes, but we can tentatively conclude that soluble, extracellular MIF likely signals through at least CD74—perhaps in concert with chemokine co-receptors to control some MDSC phenotypes associated with homing, motility and expansion (108–110). It is also conceivable—given that the CD74-binding competent MIF-P2G mutant (102) and 4-IPP-inhibited MIF (103)—that intracellular MDSC MIF may be independently involved in dictating MDSC differentiation and immune-suppressive gene expression patterns.

## Dendritic Cells

Dendritic cells (DCs) are professional antigen-presenting cells (APCs) that can either promote anti-tumor immunity through presentation of tumor-associated antigens (TAAs) or drive tolerance of anti-tumor immune responses by promoting T cell anergy (111). Several studies investigating potential roles for MIF in DC-dependent anti-tumor immunity suggest that MIF functionally impairs the ability of DCs to present TAAs to T cells leading to a dampening of anti-tumor responses.

One such study found that loss of tumor-derived MIF significantly enhances DC tumor accumulation, effector functions and, anti-tumor immunity (112). Knockdown of MIF in 4T1 cells increased intratumoral infiltration of CD11c^+^/CD8^+^/CD103^+^ DCs and increased the expression of DC co-stimulatory CD40 and CD86 receptors. This phenotype corresponded to increased CD4^+^ and CD8^+^ T cell tumor-infiltration and resultant increases in anti-tumor T lymphocyte IFN-γ production. The mechanism by which loss of tumor-derived MIF increased DC tumor accumulation was attributed to enhanced tumor cell cytolysis and ensuing tumor-associated antigen release (112).

Similar DC-inhibiting phenotypes associated with MIF have also been shown to regulate DC intratumoral infiltration, DC maturation, and DC migration all of which conspire to reduce T cell effector functions (112–114). In one such report, the effects of MIF on directly suppressing Th1 T cell activation was ruled out using neutralizing anti-MIF antibodies which were found to be unable to attenuate tumor supernatant-induced suppression of T cell proliferation *in vitro* (115).

Given the similarities, it is tempting to speculate that the requirement for MIF in maintaining M-MDSC differentiation/restraining spontaneous DC differentiation, described above (101), may also be participating in these observed increases in intratumoral DC functions upon loss of MIF. If true, we would anticipate that the observed increases in DC function would be accompanied by a reduction in intratumoral M-MDSCs (90, 101, 116).

DCs present antigens to CD4^+^ T cells *via* MHC-II or cross-present antigens to CD8^+^ T cells *via* MHC-I. Consistent with a MIF-dependent inhibition of antigen presentation, co-culture of purified CD8^+^ T cells with MIF-treated, tumor antigen-pulsed DCs reduced the ability of CD8^+^ to lyse tumor cells (104). Given that DCs cross-present antigens to CD8^+^ T cells *via* MHC-I (117), this finding indicates that MIF promotes immunosuppression, in part, by dampening DC cross-presentation of tumor antigens. Additionally, studies that looked at DC MHC-II expression found that MIF promotes downregulation of MHC-II (104, 112), which further suggests that MIF may also impair tumor antigen presentation of DCs to CD4^+^ T cells. Combined, these findings suggest that MIF promotes tumor-associated immune evasion by inhibiting DC infiltration, maturation, and antigen presentation; all of which are inherently required for the development of anti-tumor CD4^+^ and CD8^+^ T cell effector functions.

## Tumor-Associated Neutrophils (TANS)

Similar to TAMs, tumor-associated neutrophils (TANs) exhibit plasticity in the tumor microenvironment and can exist as various phenotypic subtypes depending on the composition and makeup of the tumor microenvironment (118). While the functional role for MIF in TAN biology has not been fully characterized, studies indicate that MIF influences TAN tumoral infiltration and TAN functional phenotypes.

In head and neck cancer (HNC) specimens, high levels of MIF correlate with CD66b (marker of granulocytes/neutrophils), lymph node metastasis, and reduced overall survival (119). Mechanistically, tumor-derived MIF promotes neutrophil chemotaxis through outside-in CXCR2 signaling and increases the production of neutrophil-derived CCL4 and MMP9 (119). MIF-induced CCL4 and MMP9 production in TANs may have tumor autonomous effects by promoting lymphangiogenesis as well as tumor-stromal remodeling (120, 121). Additionally, CCL4 can also increase tumor infiltration of a variety of immune cell types including T lymphocytes, by signaling through CCR5 (122). Whether MIF-dependent CCL4 expression in TANs has pro- or anti-tumorigenic effects likely depends on which immune cells infiltrate and how the tumor microenvironment specifically activates these cells.

In another study (please see Table 1 for a summary of noted studies used for this review and corresponding references) investigating a non-melanoma skin cancer (NMSC) mouse model induced by chronic UVB exposure (123), MIF-deficient mice exhibited reduced tumor growth that corresponded to significant reductions in dermal neutrophil infiltration and associated myeloperoxidase activity. This finding is particularly interesting as NMSC tumor initiation is largely due to enduring inflammatory responses and highlights the fact that MIF’s effect as a pro-inflammatory cytokine can also promote tumor growth in the context of chronic inflammation. Further studies employing additional time points of MIF-dependent regulating TAN biology and effector functions will likely reveal additional examples by which MIF promotes TAN-dependent tumor initiation in early-stage tumors through chronic inflammation and, likely, as tumors progress and develop, this phenotype may switch to tumor-associated MIF in driving TAN-associated immune evasion.

## Natural Killer (NK) Cells

NK cells are an innate immune subset of lymphocytes that exhibit potent anti-tumor cell activity both through their cytolytic function as well as by regulating adaptive immune responses. As such, NK cells are increasingly being utilized clinically for induction of anti-tumor immune responses (124, 125).

Cancer cells evade anti-tumor adaptive immune responses, in part, by downregulating MHC class I (MHC-I) molecules which are required for CD8^+^ cytotoxic T lymphocyte (CTL)-dependent activation and cytotoxicity. In MHC-I^lo^ tumors, anti-tumor CTL responses are lost and thus immune checkpoint inhibitor (ICI) immunotherapies are often ineffective. In contrast to CTLs, NK cells specifically recognize and lyse cells that do not express MHC-I. While substantial efforts have been made to restore MHC-I expression on tumors to allow for more effective CTL-mediated immunotherapy (126), harnessing the cytotoxic activity of NK cells in MHC-I^lo^ tumors represents a very attractive alternative to initiate or boost anti-tumor immunity (125).

One of the very first descriptions of an immune-suppressive function for MIF came from a study where an NK cell inhibitory activity was discovered in the aqueous humor (AH) of the eye which, when micro-sequenced, was identified to be MIF (127). Subsequent validation experiments found that neutralizing anti-MIF attenuated the NK inhibitory activity in the AH and purified recombinant mouse MIF recapitulated the AH NK inhibitory activity. Importantly, neither AH nor rMIF had any direct effect on CTL mediated lysis of allogeneic cells, and rMIF’s inhibition of NK activity corresponded to inhibition of perforin granule release by NK—but not CTL—cells.

Subsequent studies by the same group speculated that uveal melanoma—the most common intraocular cancer in adults (128)—may similarly utilize secreted MIF to evade NK-mediated cytolysis at the site of primary outgrowth and at sites of metastatic dissemination. Local and metastatic uveal melanoma cell lines were found to secrete copious amounts of MIF with metastatic lines averaging more than 2x the MIF secretion as primary cells and this melanoma-derived MIF was protective in two of the cell line supernatants tested.

Subsequent studies found that MIF-dependent NK cell inhibitory functions are not restricted to the aqueous humor and uveal melanoma cells of the eye. MIF overexpression in ovarian cancer cell lines also promotes immune evasion in NK cells; in this case by inducing the transcriptional downregulation of ovarian tumor target cell NKG2D that triggers NK-mediated tumor cell cytolysis (129).

An alternative mechanism for MIF-dependent inhibition of NK cell-mediated cytolysis was proposed following the fortuitous discovery that native—but not denatured —MIF is specifically recognized by a rat MHC class I monoclonal antibody and polyclonal anti-MIF antibodies bind to MHC class I (130). Recombinant MIF blocks MHC class I tetramer binding to NK cells suggesting that MIF may block NK function by competing for NK MHC class I molecules. The implication of this finding is that MIF contains an MHC class I structural motif that is capable of competing for HLA proteins thereby minimizing *de novo* NK cytolytic activities. Interestingly and perhaps relatedly, the cognate MIF receptor, CD74, is also known as the MHC class II invariant chain (24), which transports MHC class II molecules from the ER to the Golgi to allow for efficient late endosome peptide loading onto class II proteins (131–133). Finally, a recombinant HLA-DR protein was recently developed that was found to act by binding with high affinity to cell surface-associated CD74 which serves to actively disrupt MIF binding resulting, ultimately, in significantly reduced disease severity in models of experimental autoimmune encephalomyelitis (134).

## T Lymphocytes

T lymphocytes are essential effectors of anti-tumor immunity and are the primary targets of ICI immunotherapies (135). Multiple T lymphocyte subtypes are recruited to and infiltrate tumor stroma which, when combined, ultimately determines relative tumor immunity. These subtypes include: CD8^+^ cytotoxic T lymphocytes (CTL) (136), CD4^+^ helper T cells (137), FOXP3^+^ regulatory T cells (Tregs) (138), Th17 lymphocytes (139), and γδ T cells (140).

The first description of a functional role for MIF in T cell activation reported that murine splenocytes co-cultured with neutralizing anti-MIF antibody and activated by antigen, PMA/Ionomycin, or anti-CD3 exhibited significantly reduced IL-2 expression and concurrent reductions in T cell proliferation (7). MIF expression was further shown to be induced primarily in Th2 T lymphocyte subsets and was necessary for maximal antigen-specific antibody generation *in vivo*.

A subsequent study looking at the re-activation and relative CTL activity of *in vivo* primed splenocytes found that anti-MIF specifically *increases* CTL activity against the same irradiated syngeneic tumor cell line *in vitro* while control mAb and rMIF had no effect (141). While this study suggested that CTL-derived MIF directly inhibited anti-tumor CTL activity of primed lymphocytes, a separate finding indicated that tumor-derived MIF is similarly inhibitory to T cell activation and acts by inducing activation-induced T cell death *via* an IFN-*γ*-dependent pathway (114).

### Regulatory T cells (T_reg_)

CD4^+^CD25^+^FOXP3^+^ effector regulatory T cells (T_regs_) infiltrate into the tumor stroma as well as tumor-draining lymph nodes and potently suppress anti-tumor immunity (138, 142). Suppressive mechanisms include inhibiting IL-2 production (143), expressing/secreting immune-suppressive cytokines (e.g. IL-10, IL-35, TGF-β) (144–146), CTLA-4-dependent downregulation of co-stimulatory molecules (i.e. CD80 and CD86) on APCs (147) resulting in T cell anergy (148), ectonucleotidases that hydrolyze ATP to adenosine (149) and direct lysis of effector T cells through perforin-dependent cytotoxicity (150).

In mouse models of mammary and colon carcinoma, MIF promotes the generation/expansion of CD4^+^CD25^+^FOXP3^+^ T_regs_ by increasing IL-2 expression in activated splenocytes (151). Loss of host-derived MIF resulted in a decrease in intrasplenic and intratumoral T_regs_ following tumor inoculation and this corresponded with an increase in splenic CD4^+^ and CD8^+^ T cells and a decrease in tumor outgrowth. Interestingly, complete tumor rejection was observed in a subset of MIF^−/−^ mice that coincided with significant decreases in T_regs_ and corresponding increases in splenic CD4^+^ and CD8^+^ lymphocytes. No changes in the expression of functional T_reg_ markers (*i.e.* CTLA-4, GITR, IFN-γ, TGF-β, and IL-10) from MIF-deficient mice suggesting that MIF regulates T_reg_ *_via_*expansion/generation rather than modulation of T_reg_ effector functions.

### Gamma Delta T Cells (γδ T)

γδ T cells are a rare T cell subtype that express the γ and δ (versus α and β) chains of the T cell receptor (TCR). These cells—similar to CD4^+^ T cells—have effector functions that can either support or suppress anti-tumor immunity (140). γδ T cells that positively influence anti-tumor immunity express IFN-γ that serves to both activate antigen-experienced CD4^+^ and CD8^+^ αβ T cells (152) and increase the expression of MHC-I molecules on tumor cells (153). In this context, IFN-*γ* ^+^γδ T cells that co-express NKG2D can directly lyse cancer cells through recognition and interaction with MICA and MICB on tumor cells (154, 155). In contrast, γδ T cells that suppress anti-tumor immunity are characterized by reduced IFN-*γ* expression and high expression of interleukin 17 (IL-17) (156). γδ T17 cell-elicited IL-17 promotes tumorigenesis by increasing tumor infiltration of TAMs and MDSCs (157, 158), inducing autocrine immune-suppressive PD-L1 expression and on neighboring tumor cells (159) and directly promoting endothelial cell activation resulting in increased angiogenesis (160). In pancreatic ductal adenocarcinoma γδ T17 cells express high levels of PD-L1 resulting in the active suppression of anti-tumor αβ T cells resulting in increased tumor progression (161).

Although there are no reports of MIF driving γδ T cell effector functions in the context of tumorigenesis, MIF has been shown to regulate γδ T cell expression of IL-17 following stimulation with IL-1β and IL-23 in a mouse model of Gram-positive toxic shock syndrome (162). Interestingly, addition of recombinant MIF to lymph node-derived cells directly increases IL-17 production further supporting an amplification role for MIF in the regulation of γδ T17 cell-expressed IL-17 (163). Given that MIF can influence either pro- or anti-inflammatory phenotypes in other cell types depending on the type and severity of disease, further studies into whether, and if so how, MIF controls variable γδ T cell functions (i.e. *via* specific induction of either IFN-y, IL-17 and/or PD-L1) in models of tumor progression are needed.

### T Helper 17 (Th17) Cells

As their name implies, Th17 cells are a subset of IL-17-producing CD4^+^ helper T cells that arise when naïve CD4^+^ cells are exposed to specific cytokine signals such as TGF-β, IL-1β, and/or IL-6 and are further maintained by IL-21 and IL-23 (139). Similar to γδ T cells, Th17 lymphocytes have a variety of functions in the context of tumorigenesis (164). Pro-inflammatory Th17 responses drive colitis-induced cancer initiation/progression *via* chronic inflammation (165) while soluble IL-17-derived from Th17 cells increases tumor-associated *de novo* angiogenesis and tumor growth (166). Th17 responses have also been linked to the recruitment and immunosuppressive activity of Cd11b^+^Gr-1^+^ MDSCs (167), and suppression of CD4+ and CD8+ T effector functions *via* ectonucleotidase expression (168). In contrast, Th17-derived IL-17 is also shown to promote anti-tumor immunity by enhancing IFN-γ^+^ NK- and effector T cell activation (169) and CCL20-dependent tumor and draining lymph node infiltration of DCs resulting in increased antigen-specific activation of CTLs (170). At least some of these apparent discrepancies may be due to the differential expression of IL-17 isoforms, IL-17A *vs.* IL-17F, the site of malignancy, and other Th17-elicited cytokines (164, 171).

The first description of MIF-dependent regulation of Th17 responses came from studies showing that rMIF addition to a heterogeneous population of nodal lymphocytes results in a dramatic increase of IL-17 expression (163). Subsequent studies found that tumor-derived MIF increases the expansion and migration of anti-tumor Th17 cells in nasopharyngeal carcinoma through CXCR4 which ultimately was associated with a more favorable clinical outcome (172). Conversely, clinical analysis of circulating cytokines in women with breast cancer found that MIF expression is increased in later stage and more aggressive molecular cancer subtypes and strongly correlates with IL-17 (173). MIF has also been implicated in non-tumor associated IL-17 expression in a variety of autoimmune disorders including Hashimoto’s thyroiditis (174) and rheumatoid arthritis (RA) (175) where Th17 responses are well-characterized to drive pro-inflammation.

MIF has two functional promoter polymorphisms that increase the transcription of MIF and both are associated with susceptibility to rheumatoid arthritis (176, 177). Not coincidentally, the addition of rMIF to RA patient-derived PBMCs increases IL-17 expression to levels similar to that observed with PBMCs from an individual with a high expression MIF polymorphism haplotype (175). Finally, using a murine model of psoriasiform dermatitis induced by IL-23—a critical determinant of Th17 lymphocyte responses (178)—MIF-deficiency resulted in a dramatic reduction of disease progression (179).

## B Lymphocytes

Although not traditionally thought of as central determinants in driving tumor immunity, several recent studies implicate specific B cell subtypes and tertiary lymphoid structures (TLS) in facilitating anti-tumor immune responses and immune checkpoint blockade (ICB) efficacy in various cancer types (180–182).

In the early 1980s, Ishizaka and colleagues described a soluble polypeptide secreted by antigen-stimulated splenocytes that inhibited the *de novo* glycosylation of T cell-associated IgE-binding factor (later identified as the low-affinity IgE Fc receptor, CD23). This soluble protein was called glycosylation-inhibiting factor (GIF) for obvious reasons but the net effect of this GIF activity on decreasing CD24 glycosylation was a selective suppression of IgE synthesis on B lymphocytes (183, 184). Not coincidentally, upon purifying and micro-sequencing the isolated GIF polypeptide, it was found to be identical to the MIF amino acid sequence and when the cDNA was cloned, it was identical to the translated MIF cDNA apart from one amino acid residue in the c-terminus that was later found to be due to a technical error in the original cDNA sequencing of MIF. Interestingly, recombinant MIF has no GIF activity which led to the discovery that GIF/MIF suppressive activities on IgE antibody responses requires a post-translational modification (185) later identified as a cysteinylation of Cys-60 in GIF/MIF proteins (186).

The bulk of studies investigating MIF in B cell biology have stemmed from those evaluating the functional significance of the MIF/CD74 interaction in B cell lymphoma. Initial studies by the Shachar group showed that MIF-dependent CD74 activation drives IL-8 expression that acts to sustain chronic lymphocytic leukemia (CLL) cell survival (187). Subsequent studies found that Tap63, VLA4, and Bcl-2 are additional MIF/CD74 effectors responsible for driving CLL cell survival and homing to the bone marrow (188, 189). Less clear from these findings in B cell lymphoma is whether MIF/CD74 interactions are important in *de novo* B cell antibody responses and/or anti-tumor immunity.

Interestingly, CD74 and MIF’s non-cognate receptor (and CD74 co-receptor) CXCR4 are differentially expressed in transitional (CD19^+^CD27^-^CD38^hi^CD24^hi^) and naïve mature (CD19^+^CD27^-^CD38^int^) *vs.* activated class-switched memory (CD19^+^CD27^+^IgG^+^) B cells, which suggests that MIF may be important for the differentiation and/or activation of these B cell subsets (190). Although these findings were in the context of autoimmunity (190), the more recent findings that patients that respond to ICB treatment have a higher intratumoral frequency of un-switched and switched memory (CD19^+^CD27^+^IgD^−/+^) B cells (181) suggests that further and more detailed investigation of whether/how MIF affects B cell class switching during tumorigenesis is warranted.

All things combined, MIF influences B cell activation pathways at multiple stages of development and maturation but whether these activation phenotypes occur during tumorigenesis and extrapolate to tumor immunity is largely unexplored. Since MIF influences B cell proliferation, class switching, and cytokine production—concurrent with the seminal discoveries of the roles of B cells and TLS in ICB therapeutic efficacy—a better understanding of MIF’s role in B cell biology specifically as it relates to tumor immunity will likely reveal additional and important functional roles for MIF in governing tumor immune responses.

## Discussion

**Figure 2:**
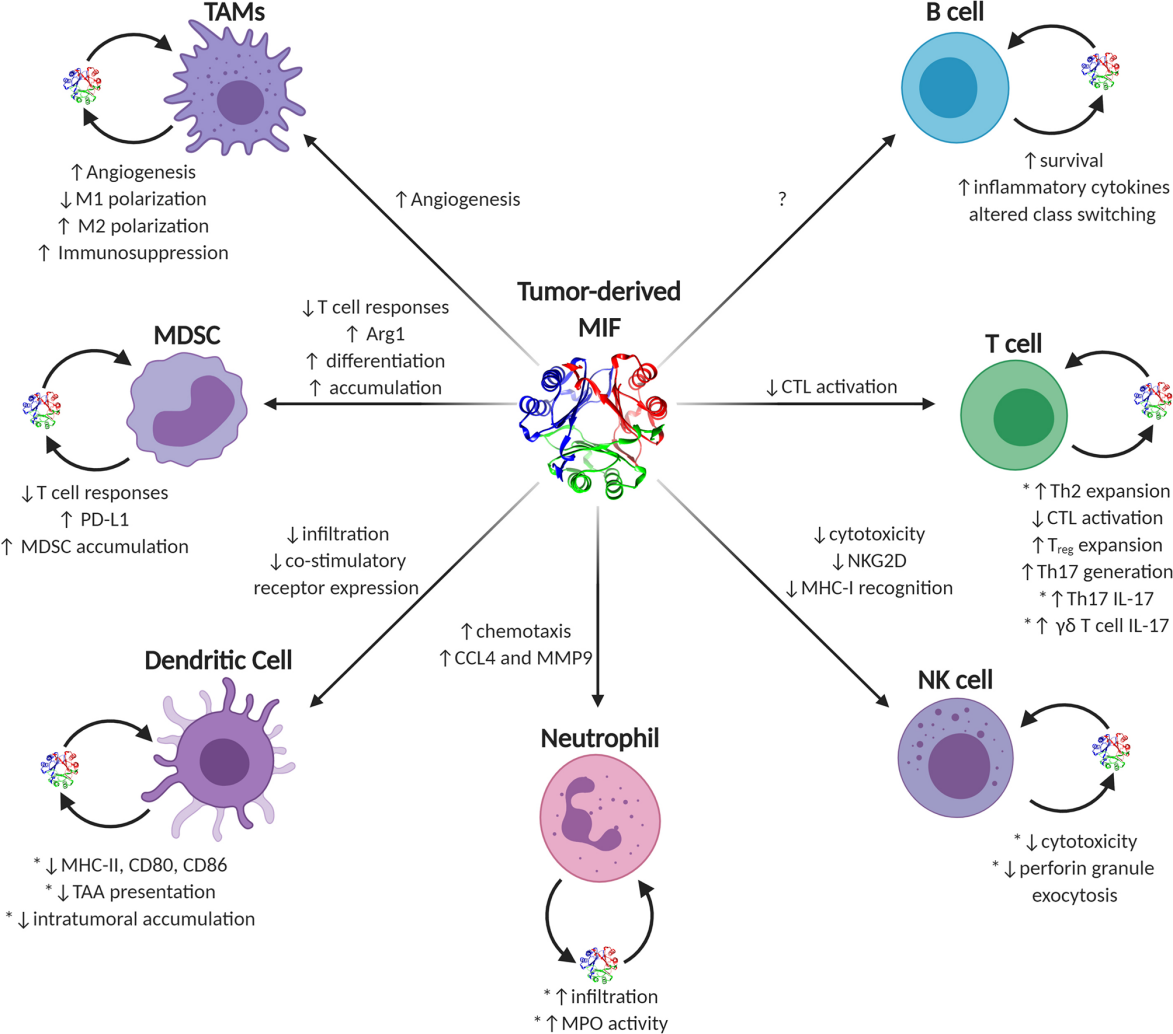
Known and putative effects of MIF on tumor-infiltrating immune cells. Graphical depiction of immune effector cells known to be influenced by MIF. Sources of intratumoral MIF include both paracrine acting tumor-secreted and autocrine acting immune cell-secreted MIF. Phenotypes ascribed to tumor-derived MIF on individual cell types are listed next to each corresponding arrow to each cell type, while autocrine-associated activities are noted next to each cell with the caveat that those activities validated using recombinant MIF sources are noted with an asterisk.

MIF is unusual among traditional cytokines. It lacks a traditional amino acid leader secretory sequence (191), it has reasonably high steady-state expression in most naïve, un-activated immune effector cells (5, 7), and it contains a very well-conserved enzymatic active site (192). MIF’s functions in eliciting either pro- or anti-tumor immune responses in individual effector cell types are numerous and often context-dependent. Generally speaking, during early tumor initiation and outgrowth, MIF supports pro-inflammatory immune phenotypes governed by early infiltrating or resident macrophages and inflammatory IL-17 producing T lymphocytes that, collectively, increase inflammation and, likely, cell and tissue damage. As tumors progress into bulkier, more immune-suppressive advanced-stage disease, MIF phenotypes begin to more closely resemble that of wound resolution activities and, in this context, MIF—both tumor cell-derived and immune effector cell-derived—switches to initiating pro-tumorigenic immune evasion and neovascular processes in a variety of immune cell types (**Figure 2**).

It is still not entirely clear how these individual MIF functions are governed and why they are frequently dependent on both the disease state and course/severity of the pathology. Multiple lines of evidence indicate that MIF’s site of action—via either cell surface receptor engagement or direct intracellular protein-protein interaction, is responsible for eliciting specific effector functions observed in individual immune cell types. One possible scenario involves a model where differential expression of CD74 and/or its various MIF co-receptors change as a result of tumor stage, immune effector cell infiltration, metabolic imbalance, and/or relative oxygenation; all of which would be expected to alter the relative phenotypic effects of soluble tumor-derived or immune effector cell-derived MIF in outside-in signaling. Less clear is whether and/or how intracellular MIF activities are differentially regulated and whether the MIF involved in these processes is from an intracellular cytosolic pool or an extracellular pool that is translocated into the cytosol (18).

An important consideration not discussed in this review is the respective role and contributions of the MIF ortholog, MIF-2, in tumor immunity. MIF-2 cooperatively signals with MIF in outside-in signaling by shared interactions with CD74 (36, 193–195), so it is plausible that where MIF-2 is co-expressed with MIF in either immune cell effectors or in tumor cells, the net effect is enhanced phenotypic tumor immunity as discussed with each of the cell types above. There is a lot to be learned about MIF-2’s differential expression in various immune effector cell types—whether MIF-2 shares similar binding activities with other MIF co-receptors and what, if any, intracellular binding proteins are shared with MIF.

Clearly MIF (and likely MIF-2) represents an intriguing and potentially clinically efficacious immunotherapeutic target for the management of malignant diseases. Several clinical trials have been undertaken and/or are currently underway investigating prospective MIF or MIF receptor inhibition in human cancers. A phase 1 study (NCT01765790) assessed the safety, pharmacokinetics, tolerability, and antitumor activity of a humanized anti-MIF monoclonal antibody (Imalumab) against solid cancers consisting primarily of colorectal carcinoma (50%), ovarian carcinoma (20%), and non-small cell lung carcinoma (NSCLC—14%) (196). Despite some toxicity issues with grade 3 allergic alveolitis, stable disease was observed in 26% of patients treated. Intriguingly, the tumor types that responded best to Imalumab were immune responsive tumors like NSCLC, ovarian, and esophageal-perhaps supporting the possibility that neutralizing MIF therapies may provide immunotherapeutic protections (196). Unfortunately, follow-up phase I/IIa and phase IIa studies were prematurely terminated for logistical reasons (poor study design and enrollment) and overall benefit-risk assessment, respectively.

An antibody targeting CD74 (Milatuzumab) underwent clinical trials (NCT00504972) and has been granted Orphan Drug Designation by the FDA for the treatment of CLL and multiple myeloma (197, 198). A CXCR4 inhibitor was previously approved to mobilize hematopoietic cells for transplantation in patients with multiple myeloma and non-Hodgkin’s lymphoma (199). Interestingly, a Phase II study (NCT02826486) is currently investigating the utility of another CXCR4 antagonist in combination with Pembrolizumab (anti-PD-1) immunotherapy for the treatment of metastatic pancreatic ductal adenocarcinoma (200).

As MIF small molecule inhibitor targeting is further refined and optimized (98, 99, 201), additional studies will explore both monotherapeutic and combination immune therapeutic targeting of MIF. Given that MIF functionally regulates the infiltration, expansion, and immune-suppressive phenotypes of such a variety of immune cell subtypes that collectively govern tumor immunity, further translational studies will be exceptionally informative.

## Author Contributions

JN reviewed literature, composed the figures and helped to write the manuscript. RM reviewed literature and wrote and organized the manuscript. All authors contributed to the article and approved the submitted version.

## Funding

This work was supported in part by NIH NCI R01CA186661 (RM), NIH (CoBRE) GB130096P3 (RM), and NIH NCI F30CA232550 (JN).

## Supplementary Material

The Supplementary Material for this article can be found online at: https://www.frontiersin.org/articles/10.3389/fimmu.2020.609948/full#supplementary-material

## Conflict of Interest

The authors declare that the research was conducted in the absence of any commercial or financial relationships that could be construed as a potential conflict of interest.

## References

[1] Gonzalez H Hagerling C Werb Z Roles of the immune system in cancer: from tumor initiation to metastatic progression. Genes Dev (2018) 32:1267–84. 10.1101/gad.314617.118 PMC6169832 30275043

[2] Bloom BR Bennett B Mechanism of a reaction in vitro associated with delayed-type hypersensitivity. Science (1966) 153:80–2. 10.1126/science.153.3731.80 5938421

[3] David JR Delayed hypersensitivity in vitro: its mediation by cell-free substances formed by lymphoid cell-antigen interaction. Proc Natl Acad Sci USA (1966) 56:72–7. 10.1073/pnas.56.1.72 PMC285677 5229858

[4] Weiser WY Temple PA Witek-Giannotti JS Remold HG Clark SC David JR Molecular cloning of a cDNA encoding a human macrophage migration inhibitory factor. Proc Natl Acad Sci USA (1989) 86:7522–6. 10.1073/pnas.86.19.7522 PMC298097 2552447

[5] Calandra T Bernhagen J Mitchell RA Bucala R The macrophage is an important and previously unrecognized source of macrophage migration inhibitory factor. J Exp Med (1994) 179:1895–902. 10.1084/jem.179.6.1895 PMC2191507 8195715

[6] Calandra T Roger T Macrophage migration inhibitory factor: a regulator of innate immunity. Nat Rev Immunol (2003) 3:791–800. 10.1038/nri1200 14502271 PMC7097468

[7] Bacher M Metz CN Calandra T Mayer K Chesney J Lohoff M An essential regulatory role for macrophage migration inhibitory factor in T-cell activation. Proc Natl Acad Sci USA (1996) 93:7849–54. 10.1073/pnas.93.15.7849 PMC38837 8755565

[8] Jankauskas SS Wong DWL Bucala R Djudjaj S Boor P Evolving complexity of MIF signaling. Cell Signal (2019) 57:76–88. 10.1016/j.cellsig.2019.01.006 30682543

[9] Lippitz BE Cytokine patterns in patients with cancer: a systematic review. Lancet Oncol (2013) 14:e218–228. 10.1016/S1470-2045(12)70582-X 23639322

[10] Koh HM Kim DC Prognostic significance of macrophage migration inhibitory factor expression in cancer patients: A systematic review and meta-analysis. Med (Baltimore) (2020) 99:e21575. 10.1097/MD.0000000000021575 PMC7592988 32769903

[11] Xiao W Dong X Zhao H Han S Nie R Zhang X Expression of MIF and c-erbB-2 in endometrial cancer. Mol Med Rep (2016) 13:3828–34. 10.3892/mmr.2016.4992 PMC4838132 26985869

[12] Tomiyasu M Yoshino I Suemitsu R Okamoto T Sugimachi K Quantification of macrophage migration inhibitory factor mRNA expression in non-small cell lung cancer tissues and its clinical significance. Clin Cancer Res (2002) 8:3755–60. 12473586

[13] Zhao YM Wang L Dai Z Wang DD Hei ZY Zhang N Validity of plasma macrophage migration inhibitory factor for diagnosis and prognosis of hepatocellular carcinoma. Int J Cancer (2011) 129:2463–72. 10.1002/ijc.25918 21213214

[14] Olsson L Lindmark G Hammarstrom ML Hammarstrom S Sitohy B Evaluating macrophage migration inhibitory factor 1 expression as a prognostic biomarker in colon cancer. Tumour Biol (2020) 42:1010428320924524. 10.1177/1010428320924524 32515296

[15] Flieger O Engling A Bucala R Lue H Nickel W Bernhagen J Regulated secretion of macrophage migration inhibitory factor is mediated by a non-classical pathway involving an ABC transporter. FEBS Lett (2003) 551:78–86. 10.1016/S0014-5793(03)00900-1 12965208

[16] Kleemann R Grell M Mischke R Zimmermann G Bernhagen J Receptor binding and cellular uptake studies of macrophage migration inhibitory factor (MIF): use of biologically active labeled MIF derivatives. J Interferon Cytokine Res (2002) 22:351–63. 10.1089/107999002753675785 12034043

[17] Xie L Qiao X Wu Y Tang J beta-Arrestin1 mediates the endocytosis and functions of macrophage migration inhibitory factor. PloS One (2011) 6:e16428. 10.1371/journal.pone.0016428 21283538 PMC3026819

[18] Kleemann R Hausser A Geiger G Mischke R Burger-Kentischer A Flieger O Intracellular action of the cytokine MIF to modulate AP-1 activity and the cell cycle through Jab1. Nature (2000) 408:211–6. 10.1038/35041591 11089976

[19] Stark K Eckart A Haidari S Tirniceriu A Lorenz M von Bruhl ML Capillary and arteriolar pericytes attract innate leukocytes exiting through venules and ‘instruct’ them with pattern-recognition and motility programs. Nat Immunol (2013) 14:41–51. 10.1038/ni.2477 23179077

[20] Bernhagen J Calandra T Mitchell RA Martin SB Tracey KJ Voelter W MIF is a pituitary-derived cytokine that potentiates lethal endotoxaemia. Nature (1993) 365:756–9. 10.1038/365756a0 8413654

[21] Bacher M Meinhardt A Lan HY Mu W Metz CN Chesney JA Migration inhibitory factor expression in experimentally induced endotoxemia. Am J Pathol (1997) 150:235–46. PMC1858503 9006339

[22] Fukuzawa J Nishihira J Hasebe N Haneda T Osaki J Saito T Contribution of macrophage migration inhibitory factor to extracellular signal-regulated kinase activation by oxidative stress in cardiomyocytes. J Biol Chem (2002) 277:24889–95. 10.1074/jbc.M112054200 11978785

[23] Mitchell RA Metz CN Peng T Bucala R Sustained mitogen-activated protein kinase (MAPK) and cytoplasmic phospholipase A2 activation by macrophage migration inhibitory factor (MIF). Regulatory role in cell proliferation and glucocorticoid action. J Biol Chem (1999) 274:18100–6. 10.1074/jbc.274.25.18100 10364264

[24] Leng L Metz CN Fang Y Xu J Donnelly S Baugh J MIF signal transduction initiated by binding to CD74. J Exp Med (2003) 197:1467–76. 10.1084/jem.20030286 PMC2193907 12782713

[25] Shi X Leng L Wang T Wang W Du X Li J CD44 is the signaling component of the macrophage migration inhibitory factor-CD74 receptor complex. Immunity (2006) 25:595–606. 10.1016/j.immuni.2006.08.020 17045821 PMC3707630

[26] Bernhagen J Krohn R Lue H Gregory JL Zernecke A Koenen RR MIF is a noncognate ligand of CXC chemokine receptors in inflammatory and atherogenic cell recruitment. Nat Med (2007) 13:587–96. 10.1038/nm1567 17435771

[27] Alampour-Rajabi S El Bounkari O Rot A Muller-Newen G Bachelerie F Gawaz M MIF interacts with CXCR7 to promote receptor internalization, ERK1/2 and ZAP-70 signaling, and lymphocyte chemotaxis. FASEB J (2015) 29:4497–511. 10.1096/fj.15-273904 26139098

[28] Tarnowski M Grymula K Liu R Tarnowska J Drukala J Ratajczak J Macrophage migration inhibitory factor is secreted by rhabdomyosarcoma cells, modulates tumor metastasis by binding to CXCR4 and CXCR7 receptors and inhibits recruitment of cancer-associated fibroblasts. Mol Cancer Res (2010) 8:1328–43. 10.1158/1541-7786.MCR-10-0288 PMC2974061 20861157

[29] Wang L Zheng JN Pei DS The emerging roles of Jab1/CSN5 in cancer. Med Oncol (2016) 33:90. 10.1007/s12032-016-0805-1 27412572

[30] Cope GA Deshaies RJ COP9 signalosome: a multifunctional regulator of SCF and other cullin-based ubiquitin ligases. Cell (2003) 114:663–71. 10.1016/S0092-8674(03)00722-0 14505567

[31] Claret FX Hibi M Dhut S Toda T Karin M A new group of conserved coactivators that increase the specificity of AP-1 transcription factors. Nature (1996) 383:453–7. 10.1038/383453a0 8837781

[32] Burger-Kentischer A Finkelmeier D Thiele M Schmucker J Geiger G Tovar GE Binding of JAB1/CSN5 to MIF is mediated by the MPN domain but is independent of the JAMM motif. FEBS Lett (2005) 579:1693–701. 10.1016/j.febslet.2005.01.080 15757663

[33] Hannemann N Jordan J Paul S Reid S Baenkler HW Sonnewald S The AP-1 Transcription Factor c-Jun Promotes Arthritis by Regulating Cyclooxygenase-2 and Arginase-1 Expression in Macrophages. J Immunol (2017) 198:3605–14. 10.4049/jimmunol.1601330 28298526

[34] Carr TM Wheaton JD Houtz GM Ciofani M JunB promotes Th17 cell identity and restrains alternative CD4(+) T-cell programs during inflammation. Nat Commun (2017) 8:301. 10.1038/s41467-017-00380-3 28824171 PMC5563507

[35] Lue H Dewor M Leng L Bucala R Bernhagen J Activation of the JNK signalling pathway by macrophage migration inhibitory factor (MIF) and dependence on CXCR4 and CD74. Cell Signal (2011) 23:135–44. 10.1016/j.cellsig.2010.08.013 PMC3586206 20807568

[36] Coleman AM Rendon BE Zhao M Qian MW Bucala R Xin D Cooperative regulation of non-small cell lung carcinoma angiogenic potential by macrophage migration inhibitory factor and its homolog, D-dopachrome tautomerase. J Immunol (2008) 181:2330–7. 10.4049/jimmunol.181.4.2330 PMC2709419 18684922

[37] Xin D Rendon BE Zhao M Winner M McGhee Coleman A Mitchell RA The MIF homologue D-dopachrome tautomerase promotes COX-2 expression through beta-catenin-dependent and -independent mechanisms. Mol Cancer Res (2010) 8:1601–9. 10.1158/1541-7786.MCR-10-0101 PMC3075601 21071513

[38] Nemajerova A Mena P Fingerle-Rowson G Moll UM Petrenko O Impaired DNA damage checkpoint response in MIF-deficient mice. EMBO J (2007) 26:987–97. 10.1038/sj.emboj.7601564 PMC1852846 17290223

[39] Winner M Koong AC Rendon BE Zundel W Mitchell RA Amplification of tumor hypoxic responses by macrophage migration inhibitory factor-dependent hypoxia-inducible factor stabilization. Cancer Res (2007) 67:186–93. 10.1158/0008-5472.CAN-06-3292 PMC2941512 17210698

[40] Winner M Leng L Zundel W Mitchell RA Macrophage migration inhibitory factor manipulation and evaluation in tumoral hypoxic adaptation. Methods Enzymol (2007) 435:355–69. 10.1016/S0076-6879(07)35018-0 PMC2943948 17998063

[41] Baugh JA Gantier M Li L Byrne A Buckley A Donnelly SC Dual regulation of macrophage migration inhibitory factor (MIF) expression in hypoxia by CREB and HIF-1. Biochem Biophys Res Commun (2006) 347:895–903. 10.1016/j.bbrc.2006.06.148 16854377

[42] Bemis L Chan DA Finkielstein CV Qi L Sutphin PD Chen X Distinct aerobic and hypoxic mechanisms of HIF-alpha regulation by CSN5. Genes Dev (2004) 18:739–44. 10.1101/gad.1180104 PMC387414 15082527

[43] Palazon A Goldrath AW Nizet V Johnson RS HIF transcription factors, inflammation, and immunity. Immunity (2014) 41:518–28. 10.1016/j.immuni.2014.09.008 PMC4346319 25367569

[44] Cortez-Retamozo V Etzrodt M Newton A Rauch PJ Chudnovskiy A Berger C Origins of tumor-associated macrophages and neutrophils. Proc Natl Acad Sci USA (2012) 109:2491–6. 10.1073/pnas.1113744109 PMC3289379 22308361

[45] Franklin RA Liao W Sarkar A Kim MV Bivona MR Liu K The cellular and molecular origin of tumor-associated macrophages. Science (2014) 344:921–5. 10.1126/science.1252510 PMC4204732 24812208

[46] Kumar V Cheng P Condamine T Mony S Languino LR McCaffrey JC CD45 Phosphatase Inhibits STAT3 Transcription Factor Activity in Myeloid Cells and Promotes Tumor-Associated Macrophage Differentiation. Immunity (2016) 44:303–15. 10.1016/j.immuni.2016.01.014 PMC4759655 26885857

[47] Mantovani A Marchesi F Malesci A Laghi L Allavena P Tumour-associated macrophages as treatment targets in oncology. Nat Rev Clin Oncol (2017) 14:399–416. 10.1038/nrclinonc.2016.217 28117416 PMC5480600

[48] Gocheva V Wang HW Gadea BB Shree T Hunter KE Garfall AL IL-4 induces cathepsin protease activity in tumor-associated macrophages to promote cancer growth and invasion. Genes Dev (2010) 24:241–55. 10.1101/gad.1874010 PMC2811826 20080943

[49] Gupta S Jain A Syed SN Snodgrass RG Pfluger-Muller B Leisegang MS IL-6 augments IL-4-induced polarization of primary human macrophages through synergy of STAT3, STAT6 and BATF transcription factors. Oncoimmunology (2018) 7:e1494110. 10.1080/2162402X.2018.1494110 30288360 PMC6169572

[50] Lin EY Nguyen AV Russell RG Pollard JW Colony-stimulating factor 1 promotes progression of mammary tumors to malignancy. J Exp Med (2001) 193:727–40. 10.1084/jem.193.6.727 PMC2193412 11257139

[51] Pyonteck SM Akkari L Schuhmacher AJ Bowman RL Sevenich L Quail DF CSF-1R inhibition alters macrophage polarization and blocks glioma progression. Nat Med (2013) 19:1264–72. 10.1038/nm.3337 PMC3840724 24056773

[52] Casazza A Laoui D Wenes M Rizzolio S Bassani N Mambretti M Impeding macrophage entry into hypoxic tumor areas by Sema3A/Nrp1 signaling blockade inhibits angiogenesis and restores antitumor immunity. Cancer Cell (2013) 24:695–709. 10.1016/j.ccr.2013.11.007 24332039

[53] Henze AT Mazzone M The impact of hypoxia on tumor-associated macrophages. J Clin Invest (2016) 126:3672–9. 10.1172/JCI84427 PMC5096805 27482883

[54] Colegio OR Chu NQ Szabo AL Chu T Rhebergen AM Jairam V Functional polarization of tumour-associated macrophages by tumour-derived lactic acid. Nature (2014) 513:559–63. 10.1038/nature13490 PMC4301845 25043024

[55] Carmona-Fontaine C Deforet M Akkari L Thompson CB Joyce JA Xavier JB Metabolic origins of spatial organization in the tumor microenvironment. Proc Natl Acad Sci USA (2017) 114:2934–9. 10.1073/pnas.1700600114 PMC5358370 28246332

[56] Qian BZ Pollard JW Macrophage diversity enhances tumor progression and metastasis. Cell (2010) 141:39–51. 10.1016/j.cell.2010.03.014 20371344 PMC4994190

[57] Murray PJ Allen JE Biswas SK Fisher EA Gilroy DW Goerdt S Macrophage activation and polarization: nomenclature and experimental guidelines. Immunity (2014) 41:14–20. 10.1016/j.immuni.2014.06.008 25035950 PMC4123412

[58] Weiskopf K Jahchan NS Schnorr PJ Cristea S Ring AM Maute RL CD47-blocking immunotherapies stimulate macrophage-mediated destruction of small-cell lung cancer. J Clin Invest (2016) 126:2610–20. 10.1172/JCI81603 PMC4922696 27294525

[59] Oldenborg PA Gresham HD Lindberg FP CD47-signal regulatory protein alpha (SIRPalpha) regulates Fcgamma and complement receptor-mediated phagocytosis. J Exp Med (2001) 193:855–62. 10.1084/jem.193.7.855 PMC2193364 11283158

[60] Chao MP Alizadeh AA Tang C Myklebust JH Varghese B Gill S Anti-CD47 antibody synergizes with rituximab to promote phagocytosis and eradicate non-Hodgkin lymphoma. Cell (2010) 142:699–713. 10.1016/j.cell.2010.07.044 20813259 PMC2943345

[61] Muraoka D Seo N Hayashi T Tahara Y Fujii K Tawara I Antigen delivery targeted to tumor-associated macrophages overcomes tumor immune resistance. J Clin Invest (2019) 129:1278–94. 10.1172/JCI97642 PMC6391090 30628894

[62] Kerkar SP Goldszmid RS Muranski P Chinnasamy D Yu Z Reger RN IL-12 triggers a programmatic change in dysfunctional myeloid-derived cells within mouse tumors. J Clin Invest (2011) 121:4746–57. 10.1172/JCI58814 PMC3226001 22056381

[63] Ghiringhelli F Apetoh L Tesniere A Aymeric L Ma Y Ortiz C Activation of the NLRP3 inflammasome in dendritic cells induces IL-1beta-dependent adaptive immunity against tumors. Nat Med (2009) 15:1170–8. 10.1038/nm.2028 19767732

[64] Tannahill GM Curtis AM Adamik J Palsson-McDermott EM McGettrick AF Goel G Succinate is an inflammatory signal that induces IL-1beta through HIF-1alpha. Nature (2013) 496:238–42. 10.1038/nature11986 PMC4031686 23535595

[65] Coussens LM Werb Z Inflammation and cancer. Nature (2002) 420:860–7. 10.1038/nature01322 PMC2803035 12490959

[66] Kryczek I Wei S Zou L Zhu G Mottram P Xu H Cutting edge: induction of B7-H4 on APCs through IL-10: novel suppressive mode for regulatory T cells. J Immunol (2006) 177:40–4. 10.4049/jimmunol.177.1.40 16785496

[67] Lin H Wei S Hurt EM Green MD Zhao L Vatan L Host expression of PD-L1 determines efficacy of PD-L1 pathway blockade-mediated tumor regression. J Clin Invest (2018) 128:805–15. 10.1172/JCI96113 PMC5785251 29337305

[68] Gordon SR Maute RL Dulken BW Hutter G George BM McCracken MN PD-1 expression by tumour-associated macrophages inhibits phagocytosis and tumour immunity. Nature (2017) 545:495–9. 10.1038/nature22396 PMC5931375 28514441

[69] Lau J Cheung J Navarro A Lianoglou S Haley B Totpal K Tumour and host cell PD-L1 is required to mediate suppression of anti-tumour immunity in mice. Nat Commun (2017) 8:14572. 10.1038/ncomms14572 28220772 PMC5321797

[70] Rodriguez PC Quiceno DG Zabaleta J Ortiz B Zea AH Piazuelo MB Arginase I production in the tumor microenvironment by mature myeloid cells inhibits T-cell receptor expression and antigen-specific T-cell responses. Cancer Res (2004) 64:5839–49. 10.1158/0008-5472.CAN-04-0465 15313928

[71] Rodriguez PC Zea AH DeSalvo J Culotta KS Zabaleta J Quiceno DG L-arginine consumption by macrophages modulates the expression of CD3 zeta chain in T lymphocytes. J Immunol (2003) 171:1232–9. 10.4049/jimmunol.171.3.1232 12874210

[72] Zea AH Rodriguez PC Atkins MB Hernandez C Signoretti S Zabaleta J Arginase-producing myeloid suppressor cells in renal cell carcinoma patients: a mechanism of tumor evasion. Cancer Res (2005) 65:3044–8. 10.1158/0008-5472.CAN-04-4505 15833831

[73] Murdoch C Muthana M Coffelt SB Lewis CE The role of myeloid cells in the promotion of tumour angiogenesis. Nat Rev Cancer (2008) 8:618–31. 10.1038/nrc2444 18633355

[74] Dirkx AE Oude Egbrink MG Wagstaff J Griffioen AW Monocyte/macrophage infiltration in tumors: modulators of angiogenesis. J Leukoc Biol (2006) 80:1183–96. 10.1189/jlb.0905495 16997855

[75] Gordon S Alternative activation of macrophages. Nat Rev Immunol (2003) 3:23–35. 10.1038/nri978 12511873

[76] Calandra T Bernhagen J Metz CN Spiegel LA Bacher M Donnelly T MIF as a glucocorticoid-induced modulator of cytokine production. Nature (1995) 377:68–71. 10.1038/377068a0 7659164

[77] Calandra T Bucala R Macrophage migration inhibitory factor: a counter-regulator of glucocorticoid action and critical mediator of septic shock. J Inflammation (1995) 47:39–51. 8913928

[78] Roger T Glauser MP Calandra T Macrophage migration inhibitory factor (MIF) modulates innate immune responses induced by endotoxin and Gram-negative bacteria. J Endotoxin Res (2001) 7:456–60. 10.1179/096805101101533089 11753217

[79] Morand EF Leech M Bernhagen J MIF: a new cytokine link between rheumatoid arthritis and atherosclerosis. Nat Rev Drug Discovery (2006) 5:399–410. 10.1038/nrd2029 16628200

[80] Mikulowska A Metz CN Bucala R Holmdahl R Macrophage migration inhibitory factor is involved in the pathogenesis of collagen type II-induced arthritis in mice. J Immunol (1997) 158:5514–7. 9164975

[81] Donnelly SC Bucala R Macrophage migration inhibitory factor: a regulator of glucocorticoid activity with a critical role in inflammatory disease. Mol Med Today (1997) 3:502–7. 10.1016/S1357-4310(97)01133-7 9430786

[82] Donnelly SC Haslett C Reid PT Grant IS Wallace WA Metz CN Regulatory role for macrophage migration inhibitory factor in acute respiratory distress syndrome. Nat Med (1997) 3:320–3. 10.1038/nm0397-320 9055860

[83] Asare Y Schmitt M Bernhagen J The vascular biology of macrophage migration inhibitory factor (MIF). Expression and effects in inflammation, atherogenesis and angiogenesis. Thromb Haemost (2013) 109:391–8. 10.1160/TH12-11-0831 23329140

[84] Bach JP Rinn B Meyer B Dodel R Bacher M Role of MIF in inflammation and tumorigenesis. Oncology (2008) 75:127–33. 10.1159/000155223 18791328

[85] Shah YM Ito S Morimura K Chen C Yim SH Haase VH Hypoxia-inducible factor augments experimental colitis through an MIF-dependent inflammatory signaling cascade. Gastroenterology (2008) 134:2036–2048, 2048 e2031-2033. 10.1053/j.gastro.2008.03.009 18439915 PMC2533811

[86] Bozza M Satoskar AR Lin G Lu B Humbles AA Gerard C Targeted disruption of migration inhibitory factor gene reveals its critical role in sepsis. J Exp Med (1999) 189:341–6. 10.1084/jem.189.2.341 PMC2192995 9892616

[87] Mitchell RA Liao H Chesney J Fingerle-Rowson G Baugh J David J Macrophage migration inhibitory factor (MIF) sustains macrophage proinflammatory function by inhibiting p53: regulatory role in the innate immune response. Proc Natl Acad Sci USA (2002) 99:345–50. 10.1073/pnas.012511599 PMC117563 11756671

[88] White ES Strom SR Wys NL Arenberg DA Non-small cell lung cancer cells induce monocytes to increase expression of angiogenic activity. J Immunol (2001) 166:7549–55. 10.4049/jimmunol.166.12.7549 11390510

[89] Wang X Chen T Leng L Fan J Cao K Duan Z MIF produced by bone marrow-derived macrophages contributes to teratoma progression after embryonic stem cell transplantation. Cancer Res (2012) 72:2867–78. 10.1158/0008-5472.CAN-11-3247 PMC3779606 22461508

[90] Yaddanapudi K Putty K Rendon BE Lamont GJ Faughn JD Satoskar A Control of tumor-associated macrophage alternative activation by macrophage migration inhibitory factor. J Immunol (2013) 190:2984–93. 10.4049/jimmunol.1201650 PMC3593945 23390297

[91] Barbosa de Souza Rizzo M Brasilino de Carvalho M Kim EJ Rendon BE Noe JT Darlene Wise A Oral squamous carcinoma cells promote macrophage polarization in an MIF-dependent manner. QJM (2018) 111:769–78. 10.1093/qjmed/hcy163 PMC6217709 30016493

[92] Chesney JA Mitchell RA Yaddanapudi K Myeloid-derived suppressor cells-a new therapeutic target to overcome resistance to cancer immunotherapy. J Leukoc Biol (2017) 102:727–40. 10.1189/jlb.5VMR1116-458RRR PMC6608049 28546500

[93] Gabrilovich D II Myeloid-Derived Suppressor Cells. Cancer Immunol Res (2017) 5:3–8. 10.1158/2326-6066.CIR-16-0297 28052991 PMC5426480

[94] Tesi RJ MDSC; the Most Important Cell You Have Never Heard Of. Trends Pharmacol Sci (2019) 40:4–7. 10.1016/j.tips.2018.10.008 30527590

[95] Simpson KD Templeton DJ Cross JV Macrophage migration inhibitory factor promotes tumor growth and metastasis by inducing myeloid-derived suppressor cells in the tumor microenvironment. J Immunol (2012) 189:5533–40. 10.4049/jimmunol.1201161 PMC3518629 23125418

[96] Otvos B Silver DJ Mulkearns-Hubert EE Alvarado AG Turaga SM Sorensen MD Cancer Stem Cell-Secreted Macrophage Migration Inhibitory Factor Stimulates Myeloid Derived Suppressor Cell Function and Facilitates Glioblastoma Immune Evasion. Stem Cells (2016) 34:2026–39. 10.1002/stem.2393 PMC5820763 27145382

[97] Alban TJ Bayik D Otvos B Rabljenovic A Leng L Jia-Shiun L Glioblastoma Myeloid-Derived Suppressor Cell Subsets Express Differential Macrophage Migration Inhibitory Factor Receptor Profiles That Can Be Targeted to Reduce Immune Suppression. Front Immunol (2020) 11:1191. 10.3389/fimmu.2020.01191 32625208 PMC7315581

[98] Cho Y Crichlow GV Vermeire JJ Leng L Du X Hodsdon ME Allosteric inhibition of macrophage migration inhibitory factor revealed by ibudilast. Proc Natl Acad Sci USA (2010) 107:11313–8. 10.1073/pnas.1002716107 PMC2895110 20534506

[99] Winner M Meier J Zierow S Rendon BE Crichlow GV Riggs R A novel, macrophage migration inhibitory factor suicide substrate inhibits motility and growth of lung cancer cells. Cancer Res (2008) 68:7253–7. 10.1158/0008-5472.CAN-07-6227 PMC2726006 18794110

[100] Oghumu S Knobloch TJ Terrazas C Varikuti S Ahn-Jarvis J Bollinger CE Deletion of macrophage migration inhibitory factor inhibits murine oral carcinogenesis: Potential role for chronic pro-inflammatory immune mediators. Int J Cancer (2016) 139:1379–90. 10.1002/ijc.30177 PMC4939094 27164411

[101] Yaddanapudi K Rendon BE Lamont G Kim EJ Al Rayyan N Richie J MIF Is Necessary for Late-Stage Melanoma Patient MDSC Immune Suppression and Differentiation. Cancer Immunol Res (2016) 4:101–12. 10.1158/2326-6066.CIR-15-0070-T PMC4740231 26603621

[102] Fingerle-Rowson G Kaleswarapu DR Schlander C Kabgani N Brocks T Reinart N A tautomerase-null macrophage migration-inhibitory factor (MIF) gene knock-in mouse model reveals that protein interactions and not enzymatic activity mediate MIF-dependent growth regulation. Mol Cell Biol (2009) 29:1922–32. 10.1128/MCB.01907-08 PMC2655604 19188446

[103] Cournia Z Leng L Gandavadi S Du X Bucala R Jorgensen WL Discovery of human macrophage migration inhibitory factor (MIF)-CD74 antagonists via virtual screening. J Med Chem (2009) 52:416–24. 10.1021/jm801100v PMC2680181 19090668

[104] Figueiredo CR Azevedo RA Mousdell S Resende-Lara PT Ireland L Santos A Blockade of MIF-CD74 Signalling on Macrophages and Dendritic Cells Restores the Antitumour Immune Response Against Metastatic Melanoma. Front Immunol (2018) 9:1132. 10.3389/fimmu.2018.01132 29875777 PMC5974174

[105] Johler SM Fuchs J Seitz G Armeanu-Ebinger S Macrophage migration inhibitory factor (MIF) is induced by cytotoxic drugs and is involved in immune escape and migration in childhood rhabdomyosarcoma. Cancer Immunol Immunother (2016) 65:1465–76. 10.1007/s00262-016-1896-4 PMC11029580 27629595

[106] Kuwahara-Ota S Shimura Y Steinebach C Isa R Yamaguchi J Nishiyama D Lenalidomide and pomalidomide potently interfere with induction of myeloid-derived suppressor cells in multiple myeloma. Br J Haematol (2020). 10.1111/bjh.16881 32558939

[107] Zhang H Ye YL Li MX Ye SB Huang WR Cai TT CXCL2/MIF-CXCR2 signaling promotes the recruitment of myeloid-derived suppressor cells and is correlated with prognosis in bladder cancer. Oncogene (2017) 36:2095–104. 10.1038/onc.2016.367 27721403

[108] Katoh H Wang D Daikoku T Sun H Dey SK Dubois RN CXCR2-expressing myeloid-derived suppressor cells are essential to promote colitis-associated tumorigenesis. Cancer Cell (2013) 24:631–44. 10.1016/j.ccr.2013.10.009 PMC3928012 24229710

[109] Han X Shi H Sun Y Shang C Luan T Wang D CXCR2 expression on granulocyte and macrophage progenitors under tumor conditions contributes to mo-MDSC generation via SAP18/ERK/STAT3. Cell Death Dis (2019) 10:598. 10.1038/s41419-019-1837-1 31395859 PMC6687752

[110] Obermajer N Muthuswamy R Odunsi K Edwards RP Kalinski P PGE(2)-induced CXCL12 production and CXCR4 expression controls the accumulation of human MDSCs in ovarian cancer environment. Cancer Res (2011) 71:7463–70. 10.1158/0008-5472.CAN-11-2449 PMC4993027 22025564

[111] Wculek SK Cueto FJ Mujal AM Melero I Krummel MF Sancho D Dendritic cells in cancer immunology and immunotherapy. Nat Rev Immunol (2020) 20:7–24. 10.1038/s41577-019-0210-z 31467405

[112] Balogh KN Templeton DJ Cross JV Macrophage Migration Inhibitory Factor protects cancer cells from immunogenic cell death and impairs anti-tumor immune responses. PloS One (2018) 13:e0197702. 10.1371/journal.pone.0197702 29864117 PMC5986154

[113] Xu S Guo X Gao X Xue H Zhang J Guo X Macrophage migration inhibitory factor enhances autophagy by regulating ROCK1 activity and contributes to the escape of dendritic cell surveillance in glioblastoma. Int J Oncol (2016) 49:2105–15. 10.3892/ijo.2016.3704 27666391

[114] Yan X Orentas RJ Johnson BD Tumor-derived macrophage migration inhibitory factor (MIF) inhibits T lymphocyte activation. Cytokine (2006) 33:188–98. 10.1016/j.cyto.2006.01.006 PMC2018658 16522371

[115] Zhou Q Yan X Gershan J Orentas RJ Johnson BD Expression of macrophage migration inhibitory factor by neuroblastoma leads to the inhibition of antitumor T cell reactivity in vivo. J Immunol (2008) 181:1877–86. 10.4049/jimmunol.181.3.1877 PMC3804024 18641325

[116] Waigel S Rendon BE Lamont G Richie J Mitchell RA Yaddanapudi K MIF inhibition reverts the gene expression profile of human melanoma cell line-induced MDSCs to normal monocytes. Genom Data (2016) 7:240–2. 10.1016/j.gdata.2015.12.025 PMC4778657 26981417

[117] Gutierrez-Martinez E Planes R Anselmi G Reynolds M Menezes S Adiko AC Cross-Presentation of Cell-Associated Antigens by MHC Class I in Dendritic Cell Subsets. Front Immunol (2015) 6:363. 10.3389/fimmu.2015.00363 26236315 PMC4505393

[118] Giese MA Hind LE Huttenlocher A Neutrophil plasticity in the tumor microenvironment. Blood (2019) 133:2159–67. 10.1182/blood-2018-11-844548 PMC6524564 30898857

[119] Dumitru CA Gholaman H Trellakis S Bruderek K Dominas N Gu X Tumor-derived macrophage migration inhibitory factor modulates the biology of head and neck cancer cells via neutrophil activation. Int J Cancer (2011) 129:859–69. 10.1002/ijc.25991 21328346

[120] Lien MY Tsai HC Chang AC Tsai MH Hua CH Wang SW Chemokine CCL4 Induces Vascular Endothelial Growth Factor C Expression and Lymphangiogenesis by miR-195-3p in Oral Squamous Cell Carcinoma. Front Immunol (2018) 9:412. 10.3389/fimmu.2018.00412 29599774 PMC5863517

[121] Quintero-Fabian S Arreola R Becerril-Villanueva E Torres-Romero JC Arana-Argaez V Lara-Riegos J Role of Matrix Metalloproteinases in Angiogenesis and Cancer. Front Oncol (2019) 9:1370. 10.3389/fonc.2019.01370 31921634 PMC6915110

[122] Vilgelm AE Richmond A Chemokines Modulate Immune Surveillance in Tumorigenesis, Metastasis, and Response to Immunotherapy. Front Immunol (2019) 10:333. 10.3389/fimmu.2019.00333 30873179 PMC6400988

[123] Martin J Duncan FJ Keiser T Shin S Kusewitt DF Oberyszyn T Macrophage migration inhibitory factor (MIF) plays a critical role in pathogenesis of ultraviolet-B (UVB) -induced nonmelanoma skin cancer (NMSC). FASEB J (2009) 23:720–30. 10.1096/fj.08-119628 18952710

[124] Morvan MG Lanier LL NK cells and cancer: you can teach innate cells new tricks. Nat Rev Cancer (2016) 16:7–19. 10.1038/nrc.2015.5 26694935

[125] Vacca P Pietra G Tumino N Munari E Mingari MC Moretta L Exploiting Human NK Cells in Tumor Therapy. Front Immunol (2019) 10:3013. 10.3389/fimmu.2019.03013 32010130 PMC6978749

[126] Garrido F Aptsiauri N Doorduijn EM Garcia Lora AM van Hall T The urgent need to recover MHC class I in cancers for effective immunotherapy. Curr Opin Immunol (2016) 39:44–51. 10.1016/j.coi.2015.12.007 26796069 PMC5138279

[127] Apte RS Sinha D Mayhew E Wistow GJ Niederkorn JY Cutting edge: role of macrophage migration inhibitory factor in inhibiting NK cell activity and preserving immune privilege. J Immunol (1998) 160:5693–6. 9637476

[128] Repp AC Mayhew ES Apte S Niederkorn JY Human uveal melanoma cells produce macrophage migration-inhibitory factor to prevent lysis by NK cells. J Immunol (2000) 165:710–5. 10.4049/jimmunol.165.2.710 10878343

[129] Krockenberger M Dombrowski Y Weidler C Ossadnik M Honig A Hausler S Macrophage migration inhibitory factor contributes to the immune escape of ovarian cancer by down-regulating NKG2D. J Immunol (2008) 180:7338–48. 10.4049/jimmunol.180.11.7338 PMC3607742 18490733

[130] Gibbings DJ Ghetu AF Dery R Befus AD Macrophage migration inhibitory factor has a MHC class I-like motif and function. Scand J Immunol (2008) 67:121–32. 10.1111/j.1365-3083.2007.02046.x 18201367

[131] Castellino F Zhong G Germain RN Antigen presentation by MHC class II molecules: invariant chain function, protein trafficking, and the molecular basis of diverse determinant capture. Hum Immunol (1997) 54:159–69. 10.1016/S0198-8859(97)00078-5 9297534

[132] Germain RN Uncovering the role of invariant chain in controlling MHC class II antigen capture. J Immunol (2011) 187:1073–5. 10.4049/jimmunol.1101663 PMC3142091 21772033

[133] Romagnoli P Germain RN The CLIP region of invariant chain plays a critical role in regulating major histocompatibility complex class II folding, transport, and peptide occupancy. J Exp Med (1994) 180:1107–13. 10.1084/jem.180.3.1107 PMC2191661 8064228

[134] Meza-Romero R Benedek G Yu X Mooney JL Dahan R Duvshani N HLA-DRalpha1 constructs block CD74 expression and MIF effects in experimental autoimmune encephalomyelitis. J Immunol (2014) 192:4164–73. 10.4049/jimmunol.1303118 PMC4028955 24683185

[135] Waldman AD Fritz JM Lenardo MJ A guide to cancer immunotherapy: from T cell basic science to clinical practice. Nat Rev Immunol (2020) 20(11):651–68. 10.1038/s41577-020-0306-5 PMC7238960 32433532

[136] Hamanishi J Mandai M Iwasaki M Okazaki T Tanaka Y Yamaguchi K Programmed cell death 1 ligand 1 and tumor-infiltrating CD8+ T lymphocytes are prognostic factors of human ovarian cancer. Proc Natl Acad Sci USA (2007) 104:3360–5. 10.1073/pnas.0611533104 PMC1805580 17360651

[137] Borst J Ahrends T Babala N Melief CJM Kastenmuller W CD4(+) T cell help in cancer immunology and immunotherapy. Nat Rev Immunol (2018) 18:635–47. 10.1038/s41577-018-0044-0 30057419

[138] Togashi Y Shitara K Nishikawa H Regulatory T cells in cancer immunosuppression - implications for anticancer therapy. Nat Rev Clin Oncol (2019) 16:356–71. 10.1038/s41571-019-0175-7 30705439

[139] Bailey SR Nelson MH Himes RA Li Z Mehrotra S Paulos CM Th17 cells in cancer: the ultimate identity crisis. Front Immunol (2014) 5:276. 10.3389/fimmu.2014.00276 24987392 PMC4060300

[140] Silva-Santos B Serre K Norell H gammadelta T cells in cancer. Nat Rev Immunol (2015) 15:683–91. 10.1038/nri3904 26449179

[141] Abe R Peng T Sailors J Bucala R Metz CN Regulation of the CTL response by macrophage migration inhibitory factor. J Immunol (2001) 166:747–53. 10.4049/jimmunol.166.2.747 11145646

[142] Zou W Regulatory T cells, tumour immunity and immunotherapy. Nat Rev Immunol (2006) 6:295–307. 10.1038/nri1806 16557261

[143] Thornton AM Shevach EM CD4+CD25+ immunoregulatory T cells suppress polyclonal T cell activation in vitro by inhibiting interleukin 2 production. J Exp Med (1998) 188:287–96. 10.1084/jem.188.2.287 PMC2212461 9670041

[144] Steinbrink K Wolfl M Jonuleit H Knop J Enk AH Induction of tolerance by IL-10-treated dendritic cells. J Immunol (1997) 159:4772–80. 9366401

[145] Collison LW Workman CJ Kuo TT Boyd K Wang Y Vignali KM The inhibitory cytokine IL-35 contributes to regulatory T-cell function. Nature (2007) 450:566–9. 10.1038/nature06306 18033300

[146] Jarnicki AG Lysaght J Todryk S Mills KH Suppression of antitumor immunity by IL-10 and TGF-beta-producing T cells infiltrating the growing tumor: influence of tumor environment on the induction of CD4+ and CD8+ regulatory T cells. J Immunol (2006) 177:896–904. 10.4049/jimmunol.177.2.896 16818744

[147] Wing K Onishi Y Prieto-Martin P Yamaguchi T Miyara M Fehervari Z CTLA-4 control over Foxp3+ regulatory T cell function. Science (2008) 322:271–5. 10.1126/science.1160062 18845758

[148] Perez VL Van Parijs L Biuckians A Zheng XX Strom TB Abbas AK Induction of peripheral T cell tolerance in vivo requires CTLA-4 engagement. Immunity (1997) 6:411–7. 10.1016/S1074-7613(00)80284-8 9133420

[149] Deaglio S Dwyer KM Gao W Friedman D Usheva A Erat A Adenosine generation catalyzed by CD39 and CD73 expressed on regulatory T cells mediates immune suppression. J Exp Med (2007) 204:1257–65. 10.1084/jem.20062512 PMC2118603 17502665

[150] Grossman WJ Verbsky JW Barchet W Colonna M Atkinson JP Ley TJ Human T regulatory cells can use the perforin pathway to cause autologous target cell death. Immunity (2004) 21:589–601. 10.1016/j.immuni.2004.09.002 15485635

[151] Choi S Kim HR Leng L Kang I Jorgensen WL Cho CS Role of macrophage migration inhibitory factor in the regulatory T cell response of tumor-bearing mice. J Immunol (2012) 189:3905–13. 10.4049/jimmunol.1102152 PMC3466372 22972922

[152] Gao Y Yang W Pan M Scully E Girardi M Augenlicht LH Gamma delta T cells provide an early source of interferon gamma in tumor immunity. J Exp Med (2003) 198:433–42. 10.1084/jem.20030584 PMC2194096 12900519

[153] Riond J Rodriguez S Nicolau ML Al Saati T Gairin JE In vivo major histocompatibility complex class I (MHCI) expression on MHCIlow tumor cells is regulated by gammadelta T and NK cells during the early steps of tumor growth. Cancer Immun (2009) 9:10. 19877577 PMC2935763

[154] Girardi M Oppenheim DE Steele CR Lewis JM Glusac E Filler R Regulation of cutaneous malignancy by gammadelta T cells. Science (2001) 294:605–9. 10.1126/science.1063916 11567106

[155] Correia DV Fogli M Hudspeth K da Silva MG Mavilio D Silva-Santos B Differentiation of human peripheral blood Vdelta1+ T cells expressing the natural cytotoxicity receptor NKp30 for recognition of lymphoid leukemia cells. Blood (2011) 118:992–1001. 10.1182/blood-2011-02-339135 21633088

[156] Fleming C Morrissey S Cai Y Yan J gammadelta T Cells: Unexpected Regulators of Cancer Development and Progression. Trends Cancer (2017) 3:561–70. 10.1016/j.trecan.2017.06.003 PMC5551453 28780933

[157] Ma S Cheng Q Cai Y Gong H Wu Y Yu X IL-17A produced by gammadelta T cells promotes tumor growth in hepatocellular carcinoma. Cancer Res (2014) 74:1969–82. 10.1158/0008-5472.CAN-13-2534 24525743

[158] Rei M Goncalves-Sousa N Lanca T Thompson RG Mensurado S Balkwill FR Murine CD27(-) Vgamma6(+) gammadelta T cells producing IL-17A promote ovarian cancer growth via mobilization of protumor small peritoneal macrophages. Proc Natl Acad Sci USA (2014) 111:E3562–3570. 10.1073/pnas.1403424111 PMC4151711 25114209

[159] Ma YF Chen C Li D Liu M Lv ZW Ji Y Targeting of interleukin (IL)-17A inhibits PDL1 expression in tumor cells and induces anticancer immunity in an estrogen receptor-negative murine model of breast cancer. Oncotarget (2017) 8:7614–24. 10.18632/oncotarget.13819 PMC5352347 27935862

[160] Wakita D Sumida K Iwakura Y Nishikawa H Ohkuri T Chamoto K Tumor-infiltrating IL-17-producing gammadelta T cells support the progression of tumor by promoting angiogenesis. Eur J Immunol (2010) 40:1927–37. 10.1002/eji.200940157 20397212

[161] Daley D Zambirinis CP Seifert L Akkad N Mohan N Werba G gammadelta T Cells Support Pancreatic Oncogenesis by Restraining alphabeta T Cell Activation. Cell (2016) 166:1485–99 e1415. 10.1016/j.cell.2016.07.046 27569912 PMC5017923

[162] Kim HK Garcia AB Siu E Tilstam P Das R Roberts S Macrophage migration inhibitory factor regulates innate gammadelta T-cell responses via IL-17 expression. FASEB J (2019) 33:6919–32. 10.1096/fj.201802433R PMC6529351 30817226

[163] Stojanovic I Cvjeticanin T Lazaroski S Stosic-Grujicic S Miljkovic D Macrophage migration inhibitory factor stimulates interleukin-17 expression and production in lymph node cells. Immunology (2009) 126:74–83. 10.1111/j.1365-2567.2008.02879.x 18624729 PMC2632697

[164] Zou W Restifo NP T(H)17 cells in tumour immunity and immunotherapy. Nat Rev Immunol (2010) 10:248–56. 10.1038/nri2742 PMC3242804 20336152

[165] Keerthivasan S Aghajani K Dose M Molinero L Khan MW Venkateswaran V beta-Catenin promotes colitis and colon cancer through imprinting of proinflammatory properties in T cells. Sci Transl Med (2014) 6:225ra228. 10.1126/scitranslmed.3007607 PMC4020714 24574339

[166] Numasaki M Fukushi J Ono M Narula SK Zavodny PJ Kudo T Interleukin-17 promotes angiogenesis and tumor growth. Blood (2003) 101:2620–7. 10.1182/blood-2002-05-1461 12411307

[167] Chang SH Mirabolfathinejad SG Katta H Cumpian AM Gong L Caetano MS T helper 17 cells play a critical pathogenic role in lung cancer. Proc Natl Acad Sci USA (2014) 111:5664–9. 10.1073/pnas.1319051111 PMC3992670 24706787

[168] Chalmin F Mignot G Bruchard M Chevriaux A Vegran F Hichami A Stat3 and Gfi-1 transcription factors control Th17 cell immunosuppressive activity via the regulation of ectonucleotidase expression. Immunity (2012) 36:362–73. 10.1016/j.immuni.2011.12.019 22406269

[169] Kryczek I Wei S Szeliga W Vatan L Zou W Endogenous IL-17 contributes to reduced tumor growth and metastasis. Blood (2009) 114:357–9. 10.1182/blood-2008-09-177360 PMC2714210 19289853

[170] Martin-Orozco N Muranski P Chung Y Yang XO Yamazaki T Lu S T helper 17 cells promote cytotoxic T cell activation in tumor immunity. Immunity (2009) 31:787–98. 10.1016/j.immuni.2009.09.014 PMC2787786 19879162

[171] McGeachy MJ Cua DJ Gaffen SL The IL-17 Family of Cytokines in Health and Disease. Immunity (2019) 50:892–906. 10.1016/j.immuni.2019.03.021 30995505 PMC6474359

[172] Li J Mo HY Xiong G Zhang L He J Huang ZF Tumor microenvironment macrophage inhibitory factor directs the accumulation of interleukin-17-producing tumor-infiltrating lymphocytes and predicts favorable survival in nasopharyngeal carcinoma patients. J Biol Chem (2012) 287:35484–95. 10.1074/jbc.M112.367532 PMC3471767 22893706

[173] Avalos-Navarro G Munoz-Valle JF Daneri-Navarro A Quintero-Ramos A Franco-Topete RA Moran-Mendoza AJ Circulating soluble levels of MIF in women with breast cancer in the molecular subtypes: relationship with Th17 cytokine profile. Clin Exp Med (2019) 19:385–91. 10.1007/s10238-019-00559-6 31102004

[174] Xue H Yang Y Zhang Y Song S Zhang L Ma L Macrophage migration inhibitory factor interacting with Th17 cells may be involved in the pathogenesis of autoimmune damage in Hashimoto’s thyroiditis. Mediators Inflammation (2015) 2015:621072. 10.1155/2015/621072 PMC4377496 25861163

[175] Hernandez-Palma LA Garcia-Arellano S Bucala R Llamas-Covarrubias MA De la Cruz-Mosso U Oregon-Romero E Functional MIF promoter haplotypes modulate Th17-related cytokine expression in peripheral blood mononuclear cells from control subjects and rheumatoid arthritis patients. Cytokine (2019) 115:89–96. 10.1016/j.cyto.2018.11.014 30467094

[176] Gregersen PK Bucala R Macrophage migration inhibitory factor, MIF alleles, and the genetics of inflammatory disorders: incorporating disease outcome into the definition of phenotype. Arthritis Rheum (2003) 48:1171–6. 10.1002/art.10880 12746889

[177] Radstake TR Sweep FC Welsing P Franke B Vermeulen SH Geurts-Moespot A Correlation of rheumatoid arthritis severity with the genetic functional variants and circulating levels of macrophage migration inhibitory factor. Arthritis Rheum (2005) 52:3020–9. 10.1002/art.21285 16200611

[178] Lubberts E The IL-23-IL-17 axis in inflammatory arthritis. Nat Rev Rheumatol (2015) 11:415–29. 10.1038/nrrheum.2015.53 25907700

[179] Bezdek S Leng L Busch H Mousavi S Rades D Dahlke M Macrophage Migration Inhibitory Factor (MIF) Drives Murine Psoriasiform Dermatitis. Front Immunol (2018) 9:2262. 10.3389/fimmu.2018.02262 30333830 PMC6176003

[180] Cabrita R Lauss M Sanna A Donia M Skaarup Larsen M Mitra S Tertiary lymphoid structures improve immunotherapy and survival in melanoma. Nature (2020) 577:561–5. 10.1038/s41586-019-1914-8 31942071

[181] Helmink BA Reddy SM Gao J Zhang S Basar R Thakur R B cells and tertiary lymphoid structures promote immunotherapy response. Nature (2020) 577:549–55. 10.1038/s41586-019-1922-8 PMC8762581 31942075

[182] Petitprez F de Reynies A Keung EZ Chen TW Sun CM Calderaro J B cells are associated with survival and immunotherapy response in sarcoma. Nature (2020) 577:556–60. 10.1038/s41586-019-1906-8 31942077

[183] Iwata M Huff TF Ishizaka K Modulation of the biologic activities of IgE-binding factor. V. The role of glycosylation-enhancing factor and glycosylation-inhibiting factor in determining the nature of IgE-binding factors. J Immunol (1984) 132:1286–93. 6363537

[184] Uede T Hirata F Hirashima M Ishizaka K Modulation of the biologic activities of IgE-binding factors. I. Identification of glycosylation-inhibiting factor as a fragment of lipomodulin. J Immunol (1983) 130:878–84. 6600257

[185] Liu YC Nakano T Elly C Ishizaka K Requirement of posttranslational modifications for the generation of biologic activity of glycosylation-inhibiting factor. Proc Natl Acad Sci USA (1994) 91:11227–31. 10.1073/pnas.91.23.11227 PMC45200 7972039

[186] Watarai H Nozawa R Tokunaga A Yuyama N Tomas M Hinohara A Posttranslational modification of the glycosylation inhibiting factor (GIF) gene product generates bioactive GIF. Proc Natl Acad Sci USA (2000) 97:13251–6. 10.1073/pnas.230445397 PMC27211 11069294

[187] Binsky I Haran M Starlets D Gore Y Lantner F Harpaz N IL-8 secreted in a macrophage migration-inhibitory factor- and CD74-dependent manner regulates B cell chronic lymphocytic leukemia survival. Proc Natl Acad Sci USA (2007) 104:13408–13. 10.1073/pnas.0701553104 PMC1948950 17686984

[188] Binsky I Lantner F Grabovsky V Harpaz N Shvidel L Berrebi A TAp63 regulates VLA-4 expression and chronic lymphocytic leukemia cell migration to the bone marrow in a CD74-dependent manner. J Immunol (2010) 184:4761–9. 10.4049/jimmunol.0904149 PMC3129539 20357260

[189] Shachar I Haran M The secret second life of an innocent chaperone: the story of CD74 and B cell/chronic lymphocytic leukemia cell survival. Leuk Lymphoma (2011) 52:1446–54. 10.3109/10428194.2011.565437 21417823

[190] Rijvers L Melief MJ van der Vuurst de Vries RM Stephant M van Langelaar J Wierenga-Wolf AF The macrophage migration inhibitory factor pathway in human B cells is tightly controlled and dysregulated in multiple sclerosis. Eur J Immunol (2018) 48:1861–71. 10.1002/eji.201847623 PMC6282801 30160778

[191] Bernhagen J Mitchell RA Calandra T Voelter W Cerami A Bucala R Purification, bioactivity, and secondary structure analysis of mouse and human macrophage migration inhibitory factor (MIF). Biochemistry (1994) 33:14144–55. 10.1021/bi00251a025 7947826

[192] Rosengren E Bucala R Aman P Jacobsson L Odh G Metz CN The immunoregulatory mediator macrophage migration inhibitory factor (MIF) catalyzes a tautomerization reaction. Mol Med (1996) 2:143–9. 10.1007/BF03402210 PMC2230029 8900542

[193] Brock SE Rendon BE Yaddanapudi K Mitchell RA Negative regulation of AMP-activated protein kinase (AMPK) activity by macrophage migration inhibitory factor (MIF) family members in non-small cell lung carcinomas. J Biol Chem (2012) 287:37917–25. 10.1074/jbc.M112.378299 PMC3488063 22988252

[194] Merk M Mitchell RA Endres S Bucala R D-dopachrome tautomerase (D-DT or MIF-2): doubling the MIF cytokine family. Cytokine (2012) 59:10–7. 10.1016/j.cyto.2012.03.014 PMC3367028 22507380

[195] Merk M Zierow S Leng L Das R Du X Schulte W The D-dopachrome tautomerase (DDT) gene product is a cytokine and functional homolog of macrophage migration inhibitory factor (MIF). Proc Natl Acad Sci USA (2011) 108:E577–85. 10.1073/pnas.1102941108 PMC3161582 21817065

[196] Mahalingam D Patel MR Sachdev JC Hart LL Halama N Ramanathan RK Phase I study of imalumab (BAX69), a fully human recombinant antioxidized macrophage migration inhibitory factor antibody in advanced solid tumours. Br J Clin Pharmacol (2020) 86(9):1836–48. 10.1111/bcp.14289 PMC7444762 32207164

[197] Haran M Mirkin V Braester A Harpaz N Shevetz O Shtreiter M A phase I-II clinical trial of the anti-CD74 monoclonal antibody milatuzumab in frail patients with refractory chronic lymphocytic leukaemia: A patient based approach. Br J Haematol (2018) 182:125–8. 10.1111/bjh.14726 28466956

[198] Kaufman JL Niesvizky R Stadtmauer EA Chanan-Khan A Siegel D Horne H Phase I, multicentre, dose-escalation trial of monotherapy with milatuzumab (humanized anti-CD74 monoclonal antibody) in relapsed or refractory multiple myeloma. Br J Haematol (2013) 163:478–86. 10.1111/bjh.12565 24112026

[199] Ghobrial IM Liu CJ Zavidij O Azab AK Baz R Laubach JP Phase I/II trial of the CXCR4 inhibitor plerixafor in combination with bortezomib as a chemosensitization strategy in relapsed/refractory multiple myeloma. Am J Hematol (2019) 94:1244–53. 10.1002/ajh.25627 31456261

[200] Bockorny B Semenisty V Macarulla T Borazanci E Wolpin BM Stemmer SM BL-8040, a CXCR4 antagonist, in combination with pembrolizumab and chemotherapy for pancreatic cancer: the COMBAT trial. Nat Med (2020) 26:878–85. 10.1038/s41591-020-0880-x 32451495

[201] Trivedi-Parmar V Jorgensen WL Advances and Insights for Small Molecule Inhibition of Macrophage Migration Inhibitory Factor. J Med Chem (2018) 61:8104–19. 10.1021/acs.jmedchem.8b00589 PMC6311451 29812929

