# Supplementary material for: MIF-Dependent Control of Tumor Immunity
Source: Front Immunol. 2020 Nov 25;11:609948. doi: 10.3389/fimmu.2020.609948 (PMC7724107; doi:10.3389/fimmu.2020.609948)
Supplement: Supplementary file 1 [file Table_1.pdf]

Cell Type	Effect	Reference
TAMs	Tumor-derived MIF: ↑monocyte-mediated angiogenesis	88
	Stromal-derived MIF: ↑macrophage-mediated angiogenesis	89, 90
	Stromal-derived MIF: ↑M2 macrophage polarization	90,91
MDSCs	Tumor-derived MIF: ↑M-MDSC differentiation	95, 104, 105, 106, 107
	Tumor-derived MIF: ↑MDSC-mediated immunosuppression	96, 97
	Stromal-derived MIF: ↑M-MDSC and G-MDSC immunosuppression	90, 100, 101
Dendritic Cells	Tumor-derived MIF: ↓DC infiltration and effector function	112-116
	↓DC-mediated presentation of tumor-associated antigens	104, 112
TANs	Tumor-derived MIF: ↑TAN chemotaxis and activation	119, 123
NK cells	Tumor-derived MIF: ↓NK cell cytolytic activity	127, 128, 129
	Regulating NK cell recognition of MHC molecules	130
T cells	↑Th2 T cell expansion	7
	↓CTL-mediated cytolytic activity	141
	Tumor-derived MIF: ↑activation-induced T cell death	114
	Stromal-derived MIF: ↑T_{reg} expansion	151
	↑γΔ T cell-mediated IL-17 production	162, 163
	↑Th17 T cell-mediated IL-17 production	163, 173, 174, 175, 179
	↑Th17 T cell expansion and migration	172
B cells	↓B cell immunoglobulin synthesis	183, 184
	Regulation of B cell survival	187, 188, 189
	Regulation of B cell differentiation/activation	190
